# Controlled human malaria infection with *Plasmodium falciparum* demonstrates impact of naturally acquired immunity on virulence gene expression

**DOI:** 10.1371/journal.ppat.1007906

**Published:** 2019-07-11

**Authors:** Anna Bachmann, Ellen Bruske, Ralf Krumkamp, Louise Turner, J. Stephan Wichers, Michaela Petter, Jana Held, Michael F. Duffy, B. Kim Lee Sim, Stephen L. Hoffman, Peter G. Kremsner, Bertrand Lell, Thomas Lavstsen, Matthias Frank, Benjamin Mordmüller, Egbert Tannich

**Affiliations:** 1 Department of Molecular Parasitology, Bernhard Nocht Institute for Tropical Medicine, Hamburg, Germany; 2 German Center for Infection Research (DZIF), partner site Hamburg-Borstel-Lübeck-Riems, Germany; 3 Institute of Tropical Medicine, University Hospital Tübingen, Tübingen, Germany; 4 Infectious Disease Epidemiology, Bernhard Nocht Institute for Tropical Medicine, Hamburg, Germany; 5 Centre for Medical Parasitology, University of Copenhagen, Copenhagen K, Denmark; 6 Mikrobiologisches Institut–Klinische Mikrobiologie, Immunologie und Hygiene, Universitätsklinikum Erlangen, Friedrich-Alexander-Universität (FAU) Erlangen-Nürnberg, Erlangen, Germany; 7 School of BioSciences, Bio21 Institute, University of Melbourne, Parkville, Victoria, Australia; 8 Sanaria Inc., Rockville, MD, United States of America; 9 German Center for Infection Research (DZIF), partner site Tübingen, Germany; 10 Centre de Recherches Médicales de Lambaréné (CERMEL), Lambaréné, Gabon; 11 German Center for Infection Research (DZIF), African partner institution, CERMEL, Gabon; Institute of Immunology and Infection Research, UNITED KINGDOM

## Abstract

The pathogenesis of *Plasmodium falciparum* malaria is linked to the variant surface antigen *Pf*EMP1, which mediates tethering of infected erythrocytes to the host endothelium and is encoded by approximately 60 *var* genes per parasite genome. Repeated episodes of malaria infection result in the gradual acquisition of protective antibodies against *Pf*EMP1 variants. The antibody repertoire is believed to provide a selective pressure driving the clonal expansion of parasites expressing unrecognized *Pf*EMP1 variants, however, due to the lack of experimental *in vivo* models there is only limited experimental evidence in support of this concept. To get insight into the impact of naturally acquired immunity on the expressed *var* gene repertoire early during infection we performed controlled human malaria infections of 20 adult African volunteers with life-long malaria exposure using aseptic, purified, cryopreserved *P*. *falciparum* sporozoites (Sanaria PfSPZ Challenge) and correlated serological data with *var* gene expression patterns from *ex vivo* parasites. Among the 10 African volunteers who developed patent infections, individuals with low antibody levels showed a steep rise in parasitemia accompanied by broad activation of multiple, predominantly subtelomeric *var* genes, similar to what we previously observed in naïve volunteers. In contrast, individuals with intermediate antibody levels developed asymptomatic infections and the *ex vivo* parasite populations expressed only few *var* gene variants, indicative of clonal selection. Importantly, in contrast to parasites from naïve volunteers, expression of *var* genes coding for endothelial protein C receptor (EPCR)-binding *Pf*EMP1 that are associated with severe childhood malaria was rarely detected in semi-immune adult African volunteers. Moreover, we followed *var* gene expression for up to six parasite replication cycles and demonstrated for the first time *in vivo* a shift in the dominant *var* gene variant. In conclusion, our data suggest that *P*. *falciparum* activates multiple subtelomeric *var* genes at the onset of blood stage infection facilitating rapid expansion of parasite clones which express *Pf*EMP1 variants unrecognized by the host’s immune system, thus promoting overall parasite survival in the face of host immunity.

## Author summary

Parasites of the species *Plasmodium falciparum* are responsible for the most severe form of malaria, particularly affecting children under the age of five years, and escape from the human immune response by antigenic variation. This is achieved by alteration of the host cell surface exposed protein *Pf*EMP1, which tethers infected erythrocytes to the endothelium. Gaining knowledge about *P*. *falciparum var* gene expression *in vivo* remains essential to understanding pathogenesis and immunity in humans. We can show that at the onset of the blood stage in malaria-naïve hosts the parasite population activates a large group of *var* genes simultaneously, but in individuals with high levels of naturally acquired immunity the *var* transcript pattern was narrowed to a single or very few *Pf*EMP1 variants compatible with parasite survival in the face of host immunity. Expression of *Pf*EMP1 variants associated with a particular receptor binding phenotype commonly observed in parasites causing severe malaria was absent in parasites recovered from infected individuals with prior exposure to malaria. These findings are of particular importance for our understanding of the strategy of malaria parasites to establish and maintain infections in the human host. They could not be characterized by the use of *in vitro* cultures.

## Introduction

The virulence of the malaria parasite *Plasmodium falciparum* has been linked to the ability of infected erythrocytes to adhere to a range of cell surface molecules expressed on the vascular endothelium. This phenomenon, known as sequestration, prevents the passage of infected erythrocytes through the spleen, which would otherwise remove the infected erythrocytes from the circulation and kill the parasites [1]. Sequestration is mediated by parasite proteins that are exported to the erythrocyte surface where they are exposed to the host’s immune system and induce variant-specific, anti-parasitic immune responses. *P*. *falciparum* possesses several multi-copy gene families coding for variant surface antigens (VSAs), which are characterized by extensive sequence polymorphisms. Members of the best-known VSA, the *P*. *falciparum* erythrocyte membrane protein 1 (*Pf*EMP1) family, mediate adhesion of infected erythrocytes to the vascular endothelium through their affinity for different host receptors including CD36, endothelial protein C receptor (EPCR) or placental chondroitin sulfate A (CSA) [2, 3]. Only a single *Pf*EMP1 variant is usually synthesized in each parasite. The long, variable, extracellular *Pf*EMP1 region responsible for receptor binding contains an N-terminal segment (NTS) and a variable number of different Duffy-binding like (DBL classes α-ζ and pam) and cysteine-rich inter-domain region (CIDR classes α-δ and pam) domains [4]. Nearly all *Pf*EMP1 have a semi-conserved N-terminal head structure consisting of an NTS and a DBLα-CIDR tandem domain [5]. Different types of the N-terminal CIDR domains confer mutually exclusive receptor binding phenotypes [6]. CIDRα1 domains, with the exception of those found in the *var1* pseudogene, bind EPCR [7] and this interaction has been linked in particular to cerebral malaria [8, 9]. CIDRα2–6 domains mediate CD36-binding [10], the most commonly observed binding phenotype. The ligands of the N-terminal CIDRδ and the more diverse CIDRβ and -γ domains are unknown, although an association with rosetting has been suggested [11–14]. The atypical *Pf*EMP1 variant VAR2CSA does not contain CIDR domains and binds placental CSA through its DBL domains [15].

Each parasite isolate’s repertoire of *Pf*EMP1 comprises a similar distribution of these mutually exclusive binding phenotypes, and this phenomenon can be explained through the ordered genetic organization of the *var* genes. Thus *Pf*EMP1 molecules have been grouped into four categories (A, B, C and E) according to the protein domain composition as well as the type of 5’ upstream sequence (UPSA-E), chromosomal localization, and direction of transcription of their encoding *var* genes [16–18]. Subtelomeric group A *var* genes encode *Pf*EMP1 with CIDRα1 or CIDRβ/γ/δ domains whereas telomeric group B and centromeric group C *var* genes encode *Pf*EMP1 with CD36-binding CIDRα2–6 domains. Recombination events have translocated a set of UPSB *var* genes to the subtelomere (group B/A) or centromere (group B/C). Group E describes the subtelomeric *var2csa* genes.

The *P*. *falciparum* reference strain NF54 encodes nine different group A *Pf*EMP1 including three predicted to bind EPCR and six with unknown binding properties of which three have CIDRβ/γ/δ domains and three are copies without a CIDR domain (VAR3). In addition, NF54 has one copy of the *var1* pseudogene. NF54 further encodes 37 group B *Pf*EMP1, of which two are B/A-type *Pf*EMP1, binding EPCR via their CIDRα1 domains, and 35 are predicted to bind CD36. Finally, the parasite encodes 13 group C *Pf*EMP1 predicted to bind CD36, and one VAR2CSA protein [4].

Expression of specific subsets of *var* genes has been linked to the clinical outcomes of malaria. Initial observations indicated that group A *var* gene expression correlates with severe forms of malaria in non-immune children, whereas group C *var* gene expression may be associated with mild malaria [19–25]. Expression of group B *var* genes has been associated with both severe and mild malaria cases [19, 20, 26–28]. These findings are in line with recent observations that only A- and B-type *var* genes coding for EPCR-binding proteins are more abundantly expressed during severe malaria [8, 9, 29–31]. A-type *var* genes are evolutionary more conserved whereas B- and C-type *var* genes are more diverse and also more abundant in the parasite genome. In accordance with their prevalent transcription in young children and their conservation, antibodies against A- and B/A-type *Pf*EMP1 proteins are rapidly acquired early in life and before antibodies to B- and C-type *Pf*EMP1 [32–36].

Most studies so far have investigated *var* gene expression from clinical isolates with unknown *var* gene repertoires and without knowledge of the history of the infection. These cross-sectional studies are very valuable for understanding the relationship between clinical malaria and certain *Pf*EMP1 types, but are limited with regards to explaining parasite strategies to establish infection in a new host after transmission. To understand the hierarchies in *var* gene expression in first-time infected individuals, we previously analyzed *ex vivo var* gene expression profiles in 18 non-immune volunteers infected with aseptic, purified, cryopreserved NF54 sporozoites (Sanaria PfSPZ Challenge (NF54)) [37]. All samples showed a remarkably conserved, uniform expression pattern. Although most *var* gene variants were detectable at the onset of the blood stage infection in each volunteer, transcription was predominantly from the subtelomeric located *var* gene groups A, B and the intermediate group B/A. In contrast, expression of the centrally located group C and B/C as well as of the three least polymorphic *var* gene sub-families *var1*, *var2csa* and *var3* was low or undetectable [37]. Interestingly, this pattern differed significantly from the NF54 culture that was used to generate the PfSPZ Challenge for these volunteer infections by mosquito passage. In this culture, the conserved *var2csa* gene was predominantly transcribed [37, 38]. These observations suggest that *var* transcription is reset during mosquito or liver passage, and at the early onset of *P*. *falciparum* blood infections an intrinsic *var* expression program determines the higher probability for expression of A- and B-type *Pf*EMP1 in naïve individuals [37].

In the present study, we investigated for the first time *in vivo* in a defined setting using NF54 parasites how naturally acquired immunity to *P*. *falciparum* affects *var* gene expression patterns early in infection. To this end, we analyzed the *var* transcript profiles of parasites isolated from lifelong malaria-exposed adults participating in a controlled human malaria infection (CHMI) study of PfSPZ Challenge in Gabon [39] and compared the observed patterns to the data set from malaria-naïve volunteers. We found that the *var* expression pattern at the onset of infection depended on the degree of immunity to *P*. *falciparum*. Parasites from lifelong malaria-exposed volunteers who developed malaria symptoms and had low levels of antibodies to various *P*. *falciparum* antigens showed a similar broad expression of subtelomeric B-type *var* genes as malaria-naïve individuals, although less A-type *var* genes were expressed. In contrast, parasites from asymptomatic volunteers who controlled parasitemia over up to three weeks and had high levels of pre-existing *P*. *falciparum* specific antibodies transcribed only a single or very few dominant group B and B/C *var* genes. In one asymptomatic individual the *var* gene expression dynamics could be monitored over six parasite replication cycles. This demonstrated for the first time *in vivo* a shift in the dominant *var* gene transcripts in the parasite population of an infected individual.

## Results

### *P*. *falciparum* infection kinetics of the volunteers

For this CHMI study [39] with direct venous inoculation (DVI) of PfSPZ Challenge, lifelong malaria-exposed individuals were recruited in Lambaréné, Gabon, where the prevalence of asymptomatic *P*. *falciparum* parasitemia is high due to the perennial transmission of malaria, but the local adult population rarely, if ever, develops severe malaria and has a low frequency of symptomatic, mild infections [40]. African adults were screened for hemoglobin genotype and 9 sickle cell trait carriers (HbAS) as well as 11 volunteers with hemoglobin HbAA were included (S1 Table). Five European adults without malaria history were also recruited as malaria-naïve controls (mean age: 26.8, range: 24.3–28.8). They were treated when the first thick blood smear was positive, a time when first malaria symptoms occurred in malaria-naïve volunteers [39, 41]. In malaria-experienced adults, treatment was commenced either once the thick blood smear was positive and symptoms that could be attributed to malaria were present, or if volunteers developed a parasitemia above 1,000 parasites/μl, or at the end of the study on day 28 post infection if no parasitemia developed [39]. Clinical outcome and safety data of the clinical studies have been published previously [39, 41].

**Fig 1:**
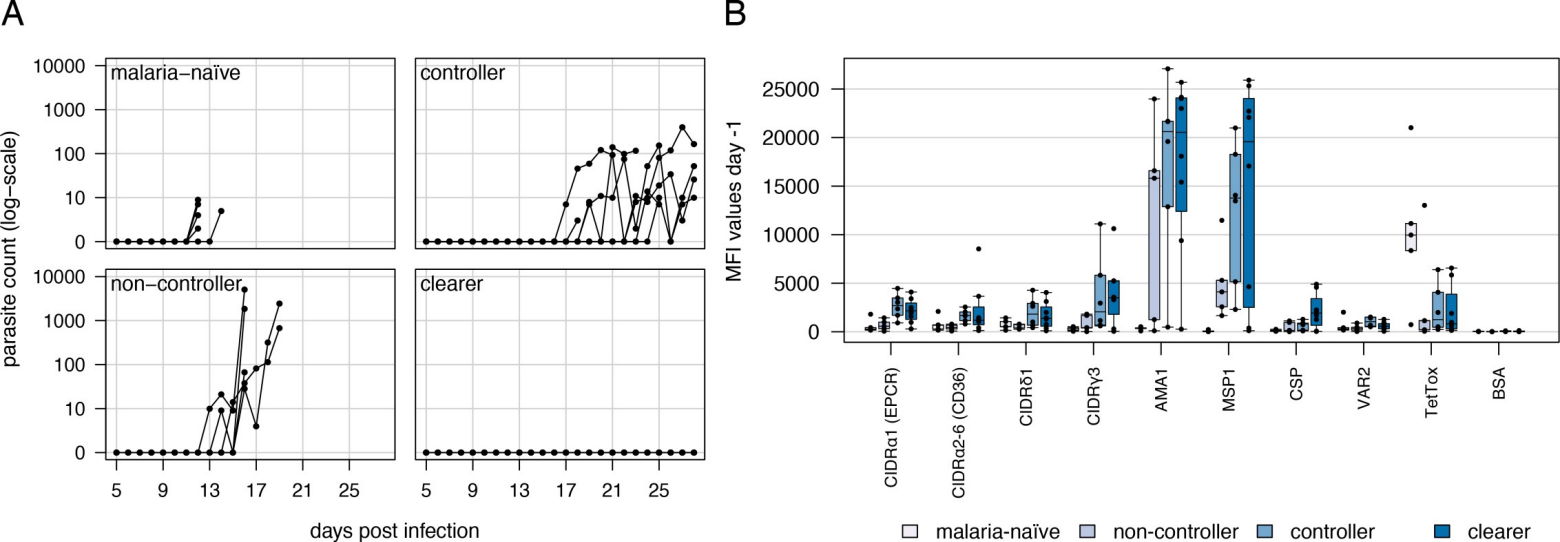
Subgrouping of volunteers into malaria-naïve, ‘non-controller’, ‘controller’ and ‘clearer’. (A) Parasite counts determined by thick blood smear. Parasite counts (parasites/μl) during the course of the CHMI trial determined by thick blood smear until treatment was initiated. Parasite replication occurs exponentially in malaria-naïve controls (n = 5), which were treated immediately after first detection of parasites in the peripheral blood. Lifelong malaria-exposed volunteers were grouped according to their ability to control parasite growth. ‘Non-controller’ (n = 6) showed a delayed exponential parasite replication, ‘controller’ (n = 6) were able to control the parasitemia below 100 parasites/μl and ‘clearer’ (n = 8) were able to eliminate injected parasites and thick blood smears were never positive for parasites. (B) Reactivity of volunteer sera with *Pf*EMP1-specific antigens and control proteins at the start of the trial. The reactivity of sera from the different volunteer groups to EPCR-binding CIDRα1 domains (n = 19), CD36-binding CIDRα2–6 domains (n = 12) and CIDR domains with unknown binding phenotype (CIDRγ3: n = 1, CIDRδ1: n = 3) was assessed by luminex assay. The *P*. *falciparum* antigens AMA1, MSP1, CSP known to induce an antibody response in humans served as positive controls. Furthermore, beads coated with VAR2CSA (VAR2), tetanus toxin (TetTox) and bovine serum albumin (BSA) were used as additional controls. Individual mean fluorescence intensities (MFI) are shown in a box plot extending from the 25^th^ to the 75^th^ percentiles with a line at the median. Number of sera analyzed from the different volunteer groups were ‘malaria-naïve’ n = 5, ‘non-controller’ n = 5, ‘controller’ n = 6 and ‘clearer’ n = 8.

Malaria-exposed adults were classified into the three groups, ‘controller’ (mean age: 22.9, range: 19.1–25.9), ‘non-controller’ (mean age: 21.8, range: 19.0–24.6) or ‘clearer’ (mean age: 21.5, range: 18.6–26.6), according to their susceptibility to infection during CHMI with PfSPZ Challenge (Fig 1A, S1 Table). Volunteers who remained negative in the thick blood smear until the end of the study on day 28 post infection, were classified as ‘clearer’ (n = 8; L1-002, L1-007, L1-009, L1-011, L1-013, L1-016, L1-021, L1-022). Volunteers who became positive after day 17 (range: 17–25) and retained low-density peak parasitemia of 14–400 parasites/μl (median: 129.5) until the day of treatment, mostly without symptoms, were classified as ‘controller’ (n = 6; L1-003, L1-010, L1-018, L1-023, L1-026, L1-028). These patients were treated between day 23–28 (median: 28). In comparison, volunteers who became parasite positive between day 13–18 (median: 15.5) and showed a exponential rise in parasitemia with median parasite counts of 1,256 parasites/μl (range: 3–5,070) at treatment initiation between day 16–19 (median: 17) were classified as ‘non-controller’ (n = 6; L1-005, L1-006, L1-008, L1-017, L1-019, L1-020). Seven HbAS carriers fell into the group of ‘clearer’ or ‘controller’ whereas two HbAS volunteers, L1-005 and L1-017, were treated on day 18 and 19 post infection, respectively, due to malaria attributed symptoms and positive thick blood smear and were accordingly classified as ‘non-controller’. Malaria-naïve European adults (L1-001, L1-014, L1-015, L1-024, L1-025) reached 2–9 parasites/μl (median: 5) between day 12 and 14 (median: 12) post infection [39]. This is comparable to 2.5–54 parasites/μl (median: 7.5) between day 10.5–15 (median: 11.5) post infection observed with 18 malaria-naïve volunteers infected with an increasing dose of PfSPZ Challenge during our previous trial in Tübingen [37, 41] (Fig 1A).

### Characterization of volunteer immune status at the start of the trial

To assess the level of anti-malarial immunity of the participants at the beginning of the study, plasma samples were screened in a Luminex assay for their reactivity to a panel of *P*. *falciparum* proteins (Fig 1B; S1 and S2 Figs; S2 and S3 Tables). This panel comprised 19 different EPCR-binding CIDRα1 domains, 12 CD36-binding CIDRα2–6 domains, and three CIDRδ1 domains from different parasite strains, as well as a single CIDRγ3 domain to analyze reactivity to *Pf*EMP1 molecules with different binding capacities (S2 Table). Several conserved and immunogenic *P*. *falciparum* antigens (apical membrane antigen 1 (AMA1), merozoite surface protein 1 (MSP1) and circumsporozoite protein (CSP)) as well as tetanus toxin were also included. MFI (mean fluorescence intensity) values representing IgG levels to these proteins are presented in Fig 1B. Malaria-naïve European volunteers showed a high recognition of tetanus toxin due to vaccination, but very low reactivity to the malaria antigens.

Using the average plus two standard deviations of the malaria-naïve IgG levels for each protein as cutoff for seropositivity, the breadth of recognition of the CIDRα1, CIDRα2–6 and CIDRδ/γ domains was calculated. This showed that both CIDRα1 and CIDRα2–6 domains but not CIDRδ/γ domains were more frequently recognized by ‘controllers’ and ‘clearers’ than by ‘non-controllers’ (Mann-Whitney U test, p = 0.0232 for CIDRα1; p = 0.0128 for CIDRα2–6; p = 0.08264 for CIDRδ/γ) (S3 Table).

IgG levels normalized to a positive control standard curve were used to assess IgG level differences between volunteer groups. IgG levels to CIDRα1 and CIDRα2–6 domains but not CIDRδ, CIDRγ or any of the merozoite antigens tested, were higher in the ‘controllers’ and ‘clearers’ compared to the ‘non-controllers’ (Mann-Whitney U test; p = 0.0139 for CIDRα1 and p = 0.0105 for CIDRα2–6, respectively). IgG levels to sporozoite antigen CSP was higher in volunteers who were able to clear the infection compared to the group of ‘non-controllers’. Altogether, these data suggest that the ability of these volunteers to control the PfSPZ infection was associated with a broader and higher level of IgG reactivity against *Pf*EMP1.

### Impact of immunity on *var* gene expression in *P*. *falciparum*

**Fig 2:**
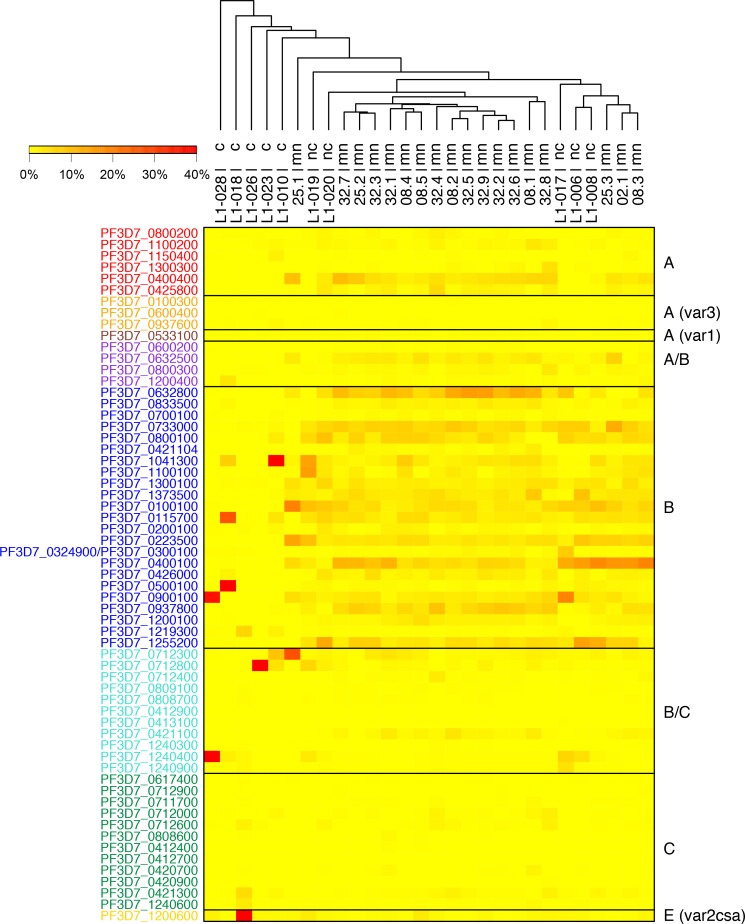
Patterns of *var* transcript level in malaria-naïve [37] and lifelong malaria-exposed adults sampled prior treatment. Heat map showing the individual *var* transcript profiles for all volunteer samples taken immediately before treatment was initiated. To correct for individual differences in the overall *var* transcript levels, the level for each *var* gene was normalized against total *var* transcript level in each sample. Hierarchical cluster analysis confirms that the subgroups of malaria-naïve (mn), ‘non-controller’ (nc) & ‘controller’ (c) volunteers are characterized by different *var* transcript patterns. Volunteers IDs of malaria-naïve samples according to [37]. Annotations of *var* gene variants are indicated on the left side, *var* gene group affiliations are indicated by the color code with A-type *var* genes in red, the subfamilies *var3* and *var1* in orange and dark red, B/A genes in purple, B-type genes in blue, B/C genes in turquoise, group C genes are colored in green and the *var2csa* gene (group E) is shown in yellow.

To understand how anti-malarial immunity influences *var* gene expression in the parasite population we analyzed the *var* transcript patterns in parasites infecting semi-immune adults and naïve volunteers. Parasites’ *var* transcript abundance profiles were determined from 10 lifelong malaria-exposed adults, five from the group of ‘non-controller’ (L1-006, L1-008, L1-017, L1-019, L1-020) and ‘controller’ (L1-010, L1-018, L1-023, L1-026, L1-028), respectively, at the day of treatment (Fig 2, S4 Table). No expression data could be generated from the five infected malaria-naïve individuals due to the low parasitemias and the small sample volume harvested during the trial. Therefore, we included our previous data set from malaria-naïve volunteers for the analysis [37] (Fig 2).

**Fig 3:**
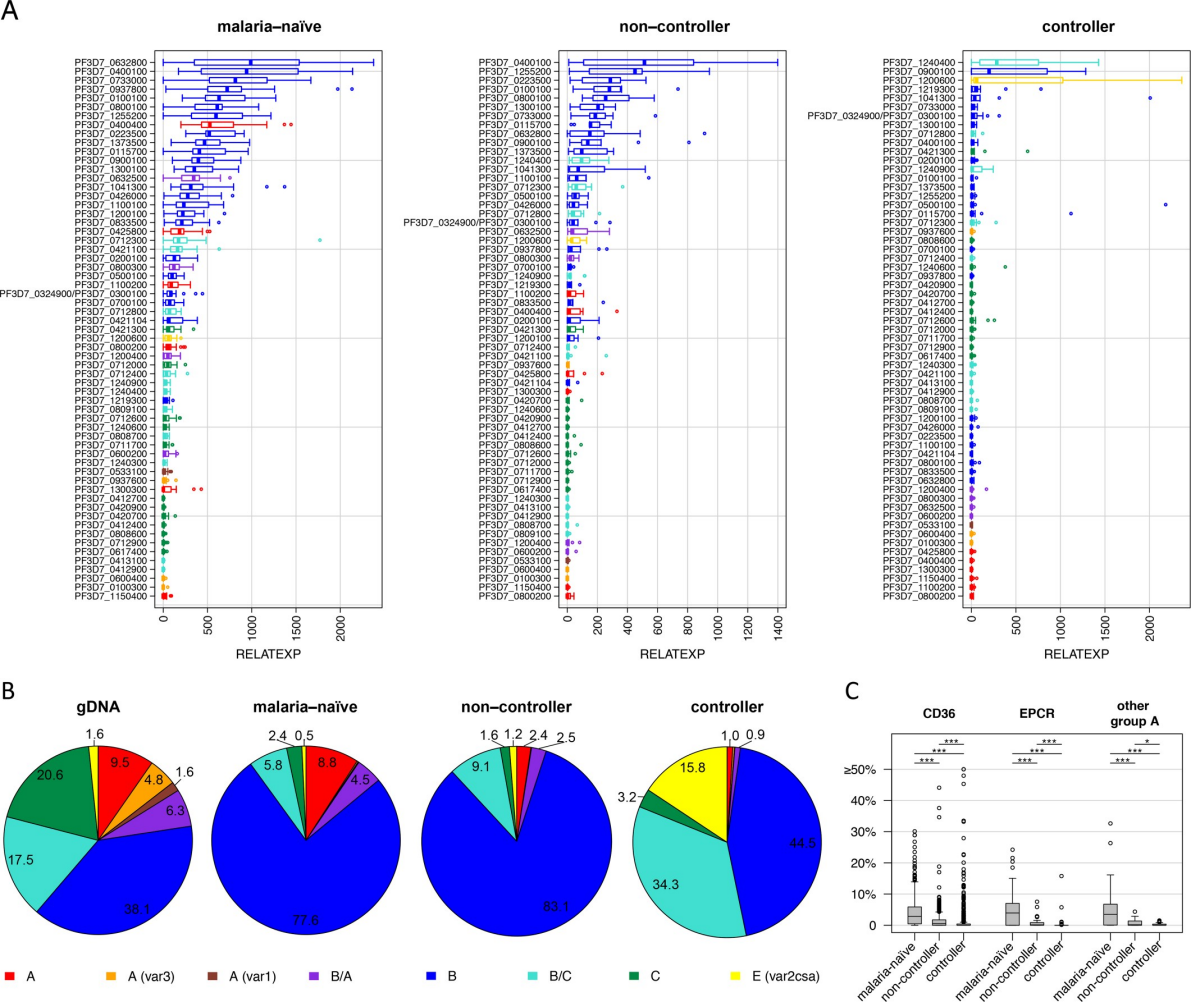
Impact of semi-immunity on parasites’ *var* gene expression. (A) Ranking of *var* genes according to transcript level detected in samples from malaria-naïve volunteers and lifelong malaria-exposed adults classified as ‘non-controller’ and ‘controller’ at the last time point sampled prior to treatment. The median *var* transcript level relative to the *sbp1* transcript level with IQR is shown for all malaria-naïve (n = 18), ‘non-controller’ (n = 5) and ‘controller’ (n = 5) volunteer samples. Expression values for genes of the different *var* groups are presented in red (group A), orange (subfamily *var3*), dark red (subfamily *var1*), purple (group B/A), blue (group B), turquoise (group B/C), green (group C) and yellow (group E). (B) Proportion of *var* transcripts according to *var* group affiliation in parasites from malaria-naïve, ‘non-controller’ & ‘controller’. For comparison, genomic proportions of genes classified into the different *var* gene groups are shown. Proportions of *var3* and *var1* type gene expression are not indicated separately, because numbers were below 0.3% in all volunteer groups. (C) Comparison of the levels of *var* transcripts encoding for EPCR-binding CIDRα1 (‘EPCR’), CD36-binding CIDRα2–6 (‘CD36’) or CIDRγ/δ (‘other group A’) domains in the different volunteer groups. Shown are expression values (%) for each *var* gene normalized against total *var* transcript level in each volunteer sample and plotted showing the median and the IQR. Gene expression of the different gene sets varied significantly between volunteer groups (Mann Whitney U test, * = p<0.05, ** = p<0.01, *** = p<0.0001).

The ‘non-controller’ samples showed broad expression of many B-type *var* genes, although most abundant variants slightly differed between each volunteer (Fig 2). Transcripts coding for A- and C-type *Pf*EMP1 proteins as well as the conserved proteins VAR2CSA, VAR1 and VAR3 were rarely detected (Fig 2, Fig 3A). In contrast, parasites from all five volunteers assigned as ‘controller’ exhibited unique expression patterns with a single or very few highly expressed *var* gene variants (Fig 2). Variants expressed at the highest level were PF10_0406/PF3D7_1041300 (L1-010), PFE0005w/PF3D7_0500100 and PFA0765c/PF3D7_0115700 (L1-018), PFI0005w/PF3D7_0900100 and PFL1955w/PF3D7_1240400 (L1-028), MAL7P1.55/PF3D7_0712800 (L1-023) and *var2csa* (L1-026). With exception of *var2csa*, all genes belong to the B- or B/C-*var* type and are only moderately expressed in parasites from malaria-naïve volunteers and ‘non-controllers’ (Fig 3A). The ‘non-controller’ expression profiles clustered together with samples from malaria-naïve volunteers and away from the ‘controllers’, indicating that the *Pf*EMP1-specific immunity observed in ‘controllers’ has a significant impact on the *var* expression profile on the population level (Fig 2). To summarize the findings, a principal component analysis (PCA) was conducted considering *var* gene expression data, MFI values obtained by luminex, parasite counts at the day of treatment and number of days until parasite positivity. This PCA confirms the separation of the volunteer samples into the two groups of ‘non-controller’ and ‘controller’ by PC1 (S3 Fig).

Interestingly, A-type *var* transcripts, particularly those encoding the EPCR-binding variant PFD0020c/PF3D7_0400400, were found to be among the most abundant in samples from malaria-naïve volunteers but were rarely detectable in samples from ‘non-controllers’ and absent in parasites infecting the group of ‘controllers’ (Fig 2, Fig 3A). Other variants detected at high levels more frequently in malaria-naïve individuals included the B-variants MAL6P1.1/PF3D7_0632800 and PFI1830c/PF3D7_0937800 as well as the B/A-variant MAL6P1.4/PF3D7_0632500. A comparison on *var* gene group level showed that transcripts of A-type *var* genes (excluding *var1* and *var3* genes) were responsible for 8.8% of the total *var* transcripts in malaria-naïve volunteers, but accounted only for 2.4% (‘non-controller’) and 1.0% (‘controller’) of the total *var* transcripts in parasites from volunteers with prior exposure to malaria (Fig 3B). Transcripts from two B/C-type *var* variants and *var2csa* were found at very high levels in parasites from volunteers able to control the infection. Therefore, the overall frequency of these two *var* gene groups was significantly elevated in the group of ‘controller’ (Fig 3B).

We next wanted to investigate whether parasites from the different groups of volunteers showed preferential expression of *var* genes associated with particular binding phenotypes. Therefore, we analyzed the levels of transcripts encoding CD36 binding *Pf*EMP1 (most B- and all C-type *var* genes), EPCR-binding *Pf*EMP1 (A-type variants PF11_0521/PF3D7_1150400, PFD0020c/PF3D7_0400400 and PFD1235w/PF3D7_0425800 as well as the B/A-variants MAL6P1.316/PF3D7_0600200 and PF08_0140/PF3D7_0800300) and other A-type *var* genes (A-type *Pf*EMP1 proteins PF11_0008/PF3D7_1100200, PF13_0003/PF3D7_1300300, PF08_0141/PF3D7_0800200) (Fig 3C). Levels of transcripts encoding EPCR-binding *Pf*EMP1 relative to the *sbp1* (skeleton binding protein 1) control gene were substantially higher in malaria-naïve than in lifelong malaria-exposed adults (Mann-Whitney U test, p<0.001) with median expression of 4.0 (range: 0.0–24.2) in the malaria-naïve group, 0.1 (range: 0.0–7.5) for ‘non-controller’ and 0.0 (range: 0–15.8) for ‘controller’. This trend was also observed for other A-type variants not predicted to bind EPCR and possibly associated with rosetting (Mann-Whitney U test, p<0.001) with a median of 3.6 (range: 0–32.8) for malaria-naïve samples and median values of 0.1 (range: 0–4.4) and 0.0 (range: 0–1.6) for samples from ‘non-controller’ and ‘controller’ (Fig 3C). Differences in median transcript levels were also observed for genes encoding CD36-binding *Pf*EMP1 between volunteers with different immune status. While malaria-naïve adults exhibited the highest transcript levels, the range of the values for ‘controller’ samples (median 0.0, range: 0–63.5) was wider than in both other groups (malaria-naïve: median 2.9, range: 0–30.1; ‘non-controller’: median 0.5, range: 0–44.1) due to a predominant expression of single individual CD36-binding variants in some of these samples.

In conclusion, *var* types previously associated with the potential to develop severe malaria were more abundantly expressed in malaria-naïve individuals than in malaria-exposed volunteers. Antibodies recognizing these *Pf*EMP1 variants are prevalent in malaria-exposed individuals and likely provide the driving force which selects the parasite population against expression of these variants.

### *Var* transcript profile changes *in vivo*

**Fig 4:**
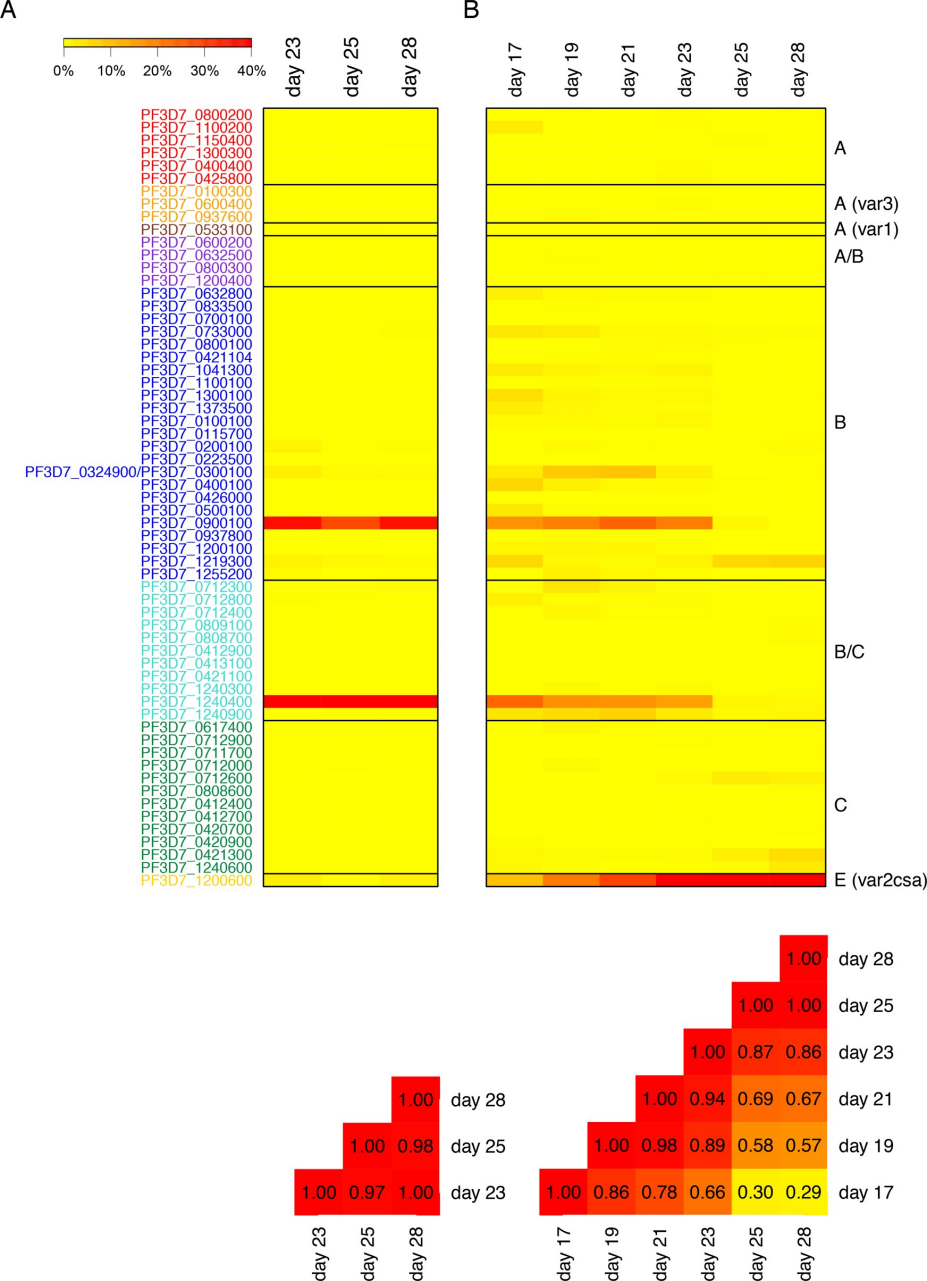
*Var* gene expression changes *in vivo*. (A, B) Monitoring of *var* gene expression over 3 or 6 parasite generations in volunteers L1-028 (A) and L1-026 (B) from the group of ‘controller’. Upper heat maps show the *var* transcript patterns of samples taken serially from volunteer L1-028 on day 13, 25 and 28 and from volunteer L1-026 on day 17, 19, 21, 23, 25 and 28 post infection. Lower heat maps present Spearman’s rank correlation coefficient (*ρ*) between the different samples. In all samples from L1-028 (A) the *var* transcript pattern was stable across the parasite replication cycles analyzed also indicated by the heat map showing the Spearman’s rank correlation coefficient between the different samples, which are all close to 1. For L1-026 (B) a major change of *var* gene expression during the time period analyzed was observed. Day of sampling is indicated as day post infection. The color scales above the expression heat map indicate the relative expression level with red representing values ≥40%, values of 0 are colored in yellow.

For several volunteers who maintained low parasitemia without malaria symptoms, we were able to collect consecutive samples over up to six replication cycles, allowing us to monitor changes in *var* profiles over time *in vivo*. Samples from two consecutive time points *in vivo* (1–3 days apart) obtained from ‘non-controller’ (L1-006, L1-017, L1-019) or ‘controller’ (L1-010) volunteers were highly correlated and showed only slight differences in their *var* gene expression profiles (*ρ*, Pearson’s correlation coefficient: L1-006: *ρ* = 0.91, L1-010: *ρ* = 0.99, L1-017: *ρ* = 0.93, L1-019: *ρ* = 0.65) (S4 Fig). Similarly, parasites from ‘controller’ L1-028 stably expressed PFI0005w/PF3D7_0900100 (B-type) and PFL1955w/PF3D7_1240400 (B/C-type) from day 23 to day 28 (5 days apart), when the study was terminated (*ρ* = 0.97–1.00) (Fig 4A). In contrast, a major change was detected during the course of the infection in samples from ‘controller’ L1-026 from who samples could be taken over 11 days. This particular volunteer was able to control the infection and maintain a parasitemia below 100 parasites/μl during the entire trial and only slight changes in the parasite counts were observed (S1 Table). From day 17, when parasites were first detected in the blood, to day 23 post infection parasites predominantly expressed three *var* gene variants: The B-type variant PFI0005w/PF3D7_0900100, the B/C-type variant PFL1955w/PF3D7_1240400 and the *var2csa* gene PFL0030c/PF3D7_1200600 (group E) (Fig 4B). On day 25 post infection the transcript abundances of another B-type variant (PFL0935c/PF3D7_1219300) started to increase and, at the same time, levels of the previously expressed B- and B/C-type variants dropped. This pattern was confirmed on day 28 post infection, when transcripts from three C-type variants (PFD1015c/PF3D7_0421300, PFL1960w/PF3D7_1240600, PF07_0051/PF3D7_0712600) were also elevated (Fig 4B). Interestingly, expression of the pregnancy-associated *var2csa* gene increased continuously during the course of the infection and, in parallel, expression of *sbp1* coding for a Maurer’s clefts protein known to be important for *Pf*EMP1 trafficking to the infected erythrocyte surface was drastically reduced (S5 Fig). In line with these observations, samples from day 17 and 28 post infection showed only weak correlation with each other (*ρ* = 0.29) (Fig 4B).

### Acquisition of *Pf*EMP1-specific antibodies during and after CHMI

To understand how CHMI affected *Pf*EMP1-specific antibody acquisition, antibody recognition of *Pf*EMP1 domains was compared before and after infection (S3 Table, S5 Table, S2 Fig). In general, malaria-naïve volunteers and ‘non-controllers’ showed higher reactivity to most antigens tested on day 28 post infection compared to pre-infection (day -1). This was also the case for a few of the ‘controllers’ and ‘clearers’. This may be due to a general unspecific polyclonal activation of the B cell response as previously observed to occur during malaria and other protozoan infections [42–46]. In attempt to identify antigen specific seroconversion, antigens to which the change in IgG response was particularly high (defined as outliners above 1.5x interquartile range of the fold changes in IgG reactivity within each volunteer) was determined (S5 Table). This showed that all malaria-naïve volunteers but only few other volunteers seroconverted to MSP1. Also malaria-naïve and ‘non-controller’ volunteers overall appeared to seroconverted more frequently to other malaria antigens compared to ‘controllers’, however the data was insufficient to reach statistical significance.

## Discussion

Despite improvements in our knowledge over the past few years, quantitative data on *var* expression patterns and transcription dynamics during infection of the human host is still sparse. This lack hampers our understanding of parasite immune evasion strategies such as antigenic variation and sequestration, neither of which can be effectively simulated *in vitro*. Previous analyses of *ex vivo* field isolate samples i) are mostly semi-quantitative and biased by the tremendous genetic variability of VSAs [21–23, 47–50], ii) are restricted to investigations of subsets of *var* genes such as promotor-based *var* subgroups or subgroups based on the *Pf*EMP1 domain cassettes (DC) architecture [4, 9, 19, 20, 29, 30, 51–53] and iii) do not provide quantitative analyses of within-host expression dynamics. To overcome these problems, we analyzed *ex vivo* samples from CHMI studies with PfSPZ Challenge generated from an NF54 cell bank. NF54 has previously been reported to consist primarily of 3D7 but an additional sibling parasite called E5 has been detected in some NF54 laboratory stocks [54, 55]. However, E5 is not present in the NF54 that was used to generate PfSPZ indicating that it is isogenic with the fully sequenced 3D7 genome strain. This enabled us to monitor RNA expression in the human host with a high-sensitivity qPCR approach for each individual *var* gene variant present in the genome of the parasite and also provide quantitative data on *var* gene expression dynamics during the course of the infection for the first time. This study also expands our previous study characterizing *in vivo var* expression patterns of parasite populations in volunteers without previous malaria exposure [37] by analyzing expression profiles in lifelong malaria-exposed African adults. We report that parasites in malaria-naïve volunteers replicate exponentially and express a broad *var* gene repertoire of mostly A- and B-type variants early after transition from hepatic to blood stage. In African volunteers termed ‘non-controller’, however, parasites show a delayed exponential parasite growth relative to naïve volunteers infected in parallel. This delay may be due to the activation of memory responses that might be reflected by the measurable level of anti-malarial antibodies at the onset of the study. Similar to malaria-naïve volunteers infected in a previous trial [37], the *var* gene expression profile in these volunteers was rather broad and dominated by B-type variants, however, A-type *var* genes were expressed less frequently than in naïve individuals. In contrast, volunteers able to control parasite growth and maintain a low parasitemia (‘controller’) appeared to have already acquired higher levels of functional antibodies against *Pf*EMP1 and other antigens during previous infections. This might result in a clonal selection of parasites within the host leading to the expression of only a single or very few *var* gene variants of B- or B/C-type. In comparison to parasites from malaria-naïve volunteers, parasites from lifelong malaria-exposed adults show more individual *var* gene expression patterns with a significant reduction of A-type variants. Similarly, genes coding for EPCR-binding *Pf*EMP1 variants were expressed less frequently in parasites from lifelong malaria-exposed Africans than in parasites infecting malaria-naïve volunteers. Particularly PFD0020c, an A-type *var* gene coding for an EPCR-binding *Pf*EMP1, was highly expressed in parasites from malaria-naïve volunteers, but transcripts were absent in parasites from almost all African volunteers. Interestingly, it has been observed that expression of group A *Pf*EMP1 is correlated with severe childhood malaria and immunity against these variants is rapidly acquired early in life before antibodies to other *Pf*EMP1 variants in malaria-exposed individuals [22, 32, 33, 35]. This may be due to a higher immunogenicity of A-type *Pf*EMP1s or their higher degree of conservation between parasite strains [22, 34–36]. Furthermore, a recent CHMI study in which Kenyan adults with varying natural exposure were infected intramuscularly with the same inoculum (PfSPZ Challenge) showed an association between the expression of *var* gene group A and DC8-like *Pf*EMP1 with low levels of antibodies to infected erythrocytes [52]. Expression of *var* genes coding for a EPCR-binding CIDRα1.1 domain were also associated with a higher parasite multiplication rate independent of the prior exposure to malaria parasites. This could indicate that parasites able to stick to the endothelium via EPCR may have a lower splenic clearance rate than parasites with other binding phenotypes and, as a result, are able to expand more rapidly [52]. Overall, expression of *var* group A and genes coding for *Pf*EMP1 with EPCR-binding CIDRα1 subtypes seems to occur more frequently in malaria-naïve individuals and in severe malaria cases [20, 51] and to increase with disease severity [29, 53]. One explanation for this higher expression of A-type and EPCR-binding variants could be an intrinsic expression program of the parasites when entering the blood phase. Additionally, more effective cytoadhesion and lower splenic clearance could explain preferential expression of A-type and EPCR binding variants on a population level.

Parasite populations from ‘controller’ volunteers with higher antibody levels against *Pf*EMP1 domains transcribed only a single or two B or B/C-type *var* variants, which were expressed at moderate levels in NF54 parasites early after liver release in malaria-naïve hosts. Presumably, each of these B or B/C-type variants represents a gap in the antibody repertoire of the infected individual. This is supported by the fact that the variants differ between almost all parasite populations infecting ‘controller’. Only parasites from L1-026 and L1-028 highly express the same two variants PFI0005w/PF3D7_0900100 and PFL1955w/PF3D7_1240400, but expression was stable between day 23 and 28 post infection in volunteer L1-028 and switched on day 25 post infection in L-026. Both proteins have the typical four domain structure of B-type *Pf*EMP1s and share most of their extracellular exposed domain subtypes: They possess an NTS domain of B3 type, a DBLα0.16, a CIDR3.4 as well as a DBLδ1 domain and diverge only in their C-terminal CIDR domain where PFI0005w/PF3D7_0900100 has a CIDRγ12 and PFL1955w/PF3D7_1240400 a CIDRβ1 domain. This implies that both proteins may share some structural or binding characteristics and may fill the same gap in the antibody repertoire.

We postulate that the different transcript levels for each *var* variant observed in non-immune hosts reflects the probability of each *var* gene to be turned on by individual parasites in the population after liver release [37] and the effect of receptor binding favoring the expansion of parasite populations transcribing specific binding phenotypes [56]. However, in semi-immune hosts parasites expressing *Pf*EMP1 variants similar to variants from previous infections are eliminated due to a memory response. In support of this ‘gap in the (antibody) wall’ theory, former studies of human *P*. *falciparum* infections have also indicated that acquisition of VSA-specific immunity can shape the VSA expression pattern, probably by gradually narrowing the repertoire of VSAs compatible with parasite survival in the semi-immune host [57–59]. This is associated with a decrease in the virulence of the infection and therefore, acquisition of VSA-specific antibodies appears to be central to acquisition of protective immunity in humans [60]. Hence, antibody levels against certain *Pf*EMP1 CIDR domain types pre-infection are to some extent predictive of the course of the infection as well as of the parasites’ *var* gene expression patterns. Identification of antigen specific IgG responses to CHMI was difficult to elucidate in part due to a generally increased IgG level to both malaria and control antigens. CHMI may induce unspecific B-cell activation [42–46], but the mechanism for this phenomenon is unknown. Most evident was an acquisition of MSP1 antibodies in malaria-naïve volunteers, but also antibody responses to a few heterologous *Pf*EMP1 antigens were observed.

Infection of malaria-naïve and semi-immune adults with *P*. *falciparum* under controlled experimental setting enables monitoring of the within-host expression dynamics longitudinally during the course of infections. To address this point, serially taken blood samples from six volunteers were analyzed in this study. The data provide the first quantitative description of an *in vivo* antigenic variation event on the level of transcription during infection with a single *P*. *falciparum* genotype. Although this was only observed in a single volunteer infected until the study was terminated by treatment, future CHMI studies with semi-immune volunteers will contribute more examples, possibly enabling the deduction of general strategies used by the parasite to escape from the human immune system and the calculation of the frequency of *var* gene switching directly from *in vivo* data. During the short time window analyzed, we could not discriminate between switching on transcriptional level initiated by the parasite and a host antibody-based selection against parasites expressing *Pf*EMP1 variants. Several studies have indicated that antigenic variation occurs in response to the appearance of anti-VSA antibodies during the course of the infection in nonhuman primate infections [61–64] as well as in a *P*. *falciparum-*infected patient [60]. Beyond that, Barnwell *et al*. have shown that *P*. *knowlesi* switches *SICAvar* variants in animals having specific antibodies, whereas the same parasite clones did not switch phenotype in naïve animals [61]. Depending on the infected individual and the antigen load, functional antibodies tend to appear 7–10 days after recognition of the antigen in the peripheral blood circulation, which would fit with the time frame in which the *var* gene transcriptional change occurred in L1-026, 7 days after positive indication by thick blood smear (day 25 after infection). To verify this, acquisition of specific antibodies against the *Pf*EMP1 variants PFI0005w/PF3D7_0900100 and PFL1955w/PF3D7_1240400 during the course of the infection should be measured, which is complicated by the fact that the exact antibody epitope is unknown and the immune response against correctly folded full-length extracellular protein regions with a size of about 200 kDa have to be analyzed. PFI0005w/PF3D7_0900100 and PFL1955w/PF3D7_1240400 as well as the variant PFL0935c/PF3D7_1219300 turned on at day 25 post infection share the CIDRα3.4 domain predicted to bind CD36 which was not represented in our luminex assay. Maybe all three *Pf*EMP1 variants share binding characteristics but differ in their epitopes recognized by the B-cell receptor and the resulting antibodies.

The observed continuous increase of *var2csa* expression supports the hypothesis that *var2csa* may act as a switching intermediate as suggested earlier [65, 66]. Ukaegbu and colleagues have shown that the highly conserved *var2csa* gene has the lowest threshold for activation after destabilization of *var* specific chromatin and could occupy this unique intermediate position within the *var* switching hierarchy [66]. Alternatively, *var2csa* could be a switch off button for *Pf*EMP1 display. Indeed, it has recently been suggested by a study analyzing *Pf*EMP1 expression in a population of Kenyan children that parasites from chronic infections may express less *Pf*EMP1 [67]. This gene can be transcriptionally turned on while being translationally repressed, a unique feature of *var2csa* amongst members of the *var* family, which would cause a shutdown of *Pf*EMP1. In line with that, transcripts from this gene have frequently been observed in non-pregnant individuals including men indicating that this gene has an alternative or additional function than ‘just’ being the ligand in pregnancy-associated malaria [68–70]. Additionally, we found that the transcript level of *sbp1* dropped during infection in L1-026. The Maurer’s cleft located protein SBP1 is generally implicated in protein export, has been shown to be essential for the display of *Pf*EMP1 on the surface of the IE [71], and was also down-regulated during severe malaria [72]. Maybe, parasites transcribing but not translating *var2csa* into a functional protein are able to survive in the host without surface-displayed *Pf*EMP1 and simultaneously down-regulate the gene expression of other components involved in *Pf*EMP1 export like the recently described novel protein complex EPIC (exported protein-interacting complex) [73]. However, further data are necessary to prove this hypothesis.

In summary, our data suggest that the parasite population exiting the liver is reset to express predominantly subtelomeric A- and, especially, B-type *var* genes, which may involve epigenetic mechanisms that specifically act on subtelomeric chromatin. In naïve individuals, parasites expressing more conserved variants with strong binding properties (e.g. to EPCR) may have a selective advantage and thus predominate in naïve infections, leading to the observed frequency of severe infections in young children. Due to the acquisition of antibodies during first infections parasites expressing these more conserved A-type variants would be cleared early in subsequent infections, resulting in the expansion of parasites expressing less conserved variants. Further acquired immunity to the more diverse variants would progressively narrow the repertoire of parasite clones that are able to establish patent and virulent infections in individuals who have been exposed to several previous malaria episodes, consistent with our observation that volunteers that were able to control parasitemia showed distinct and small repertoires of expressed *var* genes and took longer to have positive blood smears. Taken together, our study provides unique and novel insight into parasite mechanisms to establish an infection after transmission.

### Conclusion

Our results experimentally support several previously raised ideas about *var* gene expression in *P*. *falciparum* in the context of varying degrees of semi-immunity. First, antibodies against variants highly expressed in malaria-naïve hosts including A-type and EPCR-binding variants are developed first. Second, greater immunity to *Pf*EMP1 is associated with protection from infection. Third, pre-existing immunity in the host shapes the *var* expression profile of the infecting parasite population leading to expression of a single or very few variants. Moreover, a change in parasites’ *var* expression was demonstrated for the first time *in vivo* in a window of time consistent with the development of a specific immune response.

## Materials and methods

### Ethics approval and consent to participate

The Gabonese national ethics committee (Comité National d’Ethique de la Recherche) approved the study conducted in Lambaréné, which was also filed under an US FDA Investigational New Drug application (IND). Safety of participants was supervised by an independent safety review committee. The study is registered with ClinicalTrials.gov, number NCT02237586 [39]. The study was conducted according to the principles of the Declaration of Helsinki in its 6th revision as well as International Conference on Harmonization–Good Clinical Practice (ICH-GCP) guidelines. All volunteers, aged 18 to 30 years, provided written informed consent and understanding of the study and procedures was assessed with a quiz.

### CHMI trials and blood sampling

CHMI with malaria-naïve controls and lifelong malaria-exposed African adults was conducted in Lambaréné, a region with perennial transmission of malaria and semi-immunity against severe malaria in adults. Volunteers were assessed by thick blood smear and PCR for parasitemia before CHMI. In addition, all volunteers were treated with an effective antimalarial with a short half-live for 5 days in order to remove any residual parasites from the circulation [39]. During the clinical trial, healthy volunteers were infected by DVI with 3,200 aseptic, purified, cryopreserved NF54 sporozoites (PfSPZ Challenge) provided by Sanaria Inc., USA. The NF54 isolate is composed primarily of 3D7, the malaria genome reference strain, but an additional NF54 clone called “E5” has also been identified [54, 55]. To assess if E5 was present in the NF54 Sanaria strain, E5 *var* gene specific PCR was performed on DNA of the working cell bank SAN02-073009 NF54 Sanaria strain [38]. No E5-specific *var* gene fragments were amplified indicating that NF54 Sanaria is isogenic with 3D7. Blood samples for thick blood smear were taken daily from the onset of potential merozoite release at day 5 post infection and for *var* transcription profiling according to S1 Table. Briefly, 0.5–1 ml of blood pellet was recovered from Heparin or BD Vacutainer CPT tubes, washed with 5 ml of RPMI or 1xPBS and centrifuged for 5 min at 700 g. Five volumes of Trizol or Trifast Reagent (Peqlab) were added to the erythrocyte pellet and frozen at -80°C. If possible, plasma samples were taken the day before infection (-1), and on day 7, 13, 19, and 28 post infection. Non-immune volunteers received treatment with artemether-lumefantrine once their thick blood smear became parasite positive by microscopy. In African adults, treatment was administered once thick blood smear was positive and any symptoms that could be attributed to malaria were present. Volunteers with a parasitemia above 1,000 parasites/μL were treated irrespective of symptoms. All volunteers without parasitemia or remaining at low-density parasitemia without symptoms were treated on day 28 post infection [39].

### RNA purification and cDNA synthesis

RNeasy Mini (Qiagen) with on-column DNase I (Qiagen) treatment was used for RNA purification. Absence of gDNA was checked for each sample using 50 ng RNA and the *sbp1* primer set. cDNA synthesis was performed as described previously [37].

### Quantitative real-time PCR

The LightCycler 480 (Roche) was used for quantitative real-time PCR analysis using the provided software version 1.5. cDNA template was mixed with QuantiTect SYBR Green PCR reagent (Qiagen) and 0.3 μM sense and antisense primer in a final volume of 10 μl. Reactions were incubated at 95°C for 15 min, then subjected to 40 cycles of 95°C for 15 s and 60°C for 1 min and a subsequent melting step (60–95°C). The specificity of each primer pair was confirmed after each qPCR run by dissociation curve analysis. Ct calculation was done using the fit points analysis method provided by the software. Expression of *sbp1* (PFE0065w/PF3D7_0501300) was used for normalization and Ct values obtained by analysis of 2.5 ng gDNA from pre-mosquito parasites were used for calibration. Relative quantification of the NF54 *var* repertoire by 2^-ΔΔCt^ analysis was performed using previously described primer sets as well as 24 new primer sets (S6 Table) [37]. Furthermore, primer pairs targeting the housekeeping genes *fructose-bisphosphate aldolase* (PF14_0425/PF3D7_1444800) and *arginyl-tRNA synthetase* (PFL0900c/PF3D7_1218600) were included (S6 Table). Relative expression data (RELATEXP) were corrected for amplification efficiency of each primer pair, which was determined by dilution of a single gDNA from NF54 over 5–6 logs of concentration (S6 Table). Only samples with a Ct value below 30 for the two housekeeping controls *fructose-bisphosphate aldolase* and *arginyl-tRNA synthetase* were included, which was shown to be a useful standard for *var* gene transcript analysis in patient samples [37, 51]. For further control, Ct values of both housekeeping genes as well as *sbp1* (normalizer) were compared between the volunteer groups and were shown to be similar (S6 Fig). Data from our previous study with malaria-naïve volunteers were transformed so that the relative expression value was divided by two for three of the formerly used primer pairs because they amplified two *var* genes (PFD0630c/PF3D7_0412900 & PFD0635c/PF3D7_0413100, PFL1955w/PF3D7_1240400 & PFL1970w/PF3D7_1240900, PFD0995c/PF3D7_0420700 & PFD1000c/PF3D7_0420900).

Heat maps are used to display *var* gene expression on individual level. For *var* genes the relative expression per individual (i.e., percent gene expressed) was used and variants without detected Ct-values (i.e., >40 cycles) were set to 0. A cut-off of 40% was used to limit maximum expression levels in the *var* gene expression heat maps. Hierarchical clustering, with the Euclidian distance using complete linkage method, was applied for *var* gene expression in the different volunteers and the results are displayed in a dendrogram. RELATEXP distributions for *var* genes associated with particular binding phenotypes over the different volunteer groups are plotted showing the median and the interquartile range (IQR) and the non-parametric Mann-Whitney U test was applied to calculate *ρ*-values on distributions differences between volunteer groups. Pearson correlation coefficient (*ρ*) along with the 95%-confidence interval was calculated to measure linear correlation between relative expressions in individuals measured on different days post infection. Pearson correlation coefficients on the association between gene expression generations were calculated and displayed using heat maps. All analysis and graphics were done using the base package of R version 3.4.0.

### Luminex assay

35 recombinant HIS-tagged CIDR domains (S2 Table) were expressed in Drosophila Sf9 cells, and purified by nickel affinity chromatography as previously described [7, 10, 33]. Total IgG levels against these proteins were measured using a multiplex luminex assay, as previously described [74] with minor modifications. Briefly, plasma samples were diluted 1:80 in Assay Buffer E (ABE: 0.1% BSA, 0.05% Tween-20 in PBS, pH7.4). In ABE, a ten point, two-fold dilution of pooled positive plasma starting with a dilution of 1:40 was carried out. 50 μl of beads and 50 μl of diluted plasma were added to 96-well microtiter plates (MSBVS 1210, Millipore, USA) pre-wetted with ABE. 50 μl of phycoerythrin-conjugated Goat Anti-Human IgG (Jackson ImmunoResearch Laboratories), diluted 1:3500 was added and using the BioPlex^100^ system, mean fluorescent intensities and antibody concentrations were measured. Using the pooled positive plasma, a standard curve was generated with an arbitrary value of 1000 relative units assigned to the highest concentration, and IgG concentrations were interpolated from the curves to give normalized values as “observed concentrations” (obs conc).

Individual IgG responses of 5 malaria-naïve controls and 19 lifelong malaria-exposed individuals (‘non-controller’: n = 5, ‘controller’: n = 6, ‘protected’: n = 8) were measured. In total, plasma samples from day -1 (prior to infection), day 7, 13, 19 and 28 post infection were screened for individual recognition of 19 different CIDRα1, 12 CIDRα2–6, three CIDRδ1 domains and a single CIDRγ3 domain to have a widespread fishing net for antibodies reacting with all main types of *Pf*EMP1 variants, i.e. those mediating binding to EPCR, CD36 or other unknown receptors (S2 Table). The merozoite antigens AMA1 and MSP1, the sporozoite protein CSP as well as tetanus toxin served as positive controls, VAR2CSA and BSA were used as negative controls. Plasma from volunteer L1-020 (‘non-controller’, male) were excluded from analysis due to an irregular high reactivity against almost all antigens tested at all time points tested. To analyze the acquisition of antibodies during infection, the obs conc ratio between day -1 before and day 28 after infection was calculated for all volunteers except L1-025 and L1-028 for whom no day 28 sera were available.

### Principal component analysis

Principal component analysis (PCA) transforms correlated measurements into a set of uncorrelated values (so called principal components) by summarizing covariance measured in different directions, hence simplifying the underlying data structure. The first component accounts for most data variance, while the consecutive components summarise as much of the remaining variance as possible. For our model *var* gene expression data, MFI values, parasite counts at the day of treatment, and number days until positivity were used as standardized (N[0,1]) measurements. Only data from ‘controller’ and ‘non-controller’ were considered. A scatterplot was drawn to show clustering of participants over the study data, where individual scores of the first two principal components were plotted, which account for most data variance.

## Supporting information

S1 Table Overview of volunteer characteristics and parasite counts (parasites/μl) determined by thick blood smear from day 12 to day 28 post infection. (DOCX)

S2 Table Proteins on luminex plex. (DOCX)

S3 Table Breadth of IgG recognition of CIDR domains for each donor. For each donor at day -1 and 28, the proportion (%) of CIDRα2–6 (n = 12), CIDRα1 (n = 19) and CIDRδ/γ (n = 4) domains recognized at IgG levels above the mean plus two standard deviations of the IgG levels of the naïve donors at day -1, is listed. nd = not determined. * day 19
(DOCX)

S4 Table Relative expression (RELATEXP) measured by qPCR for all volunteer samples. (XLSX)

S5 Table Change in IgG levels from day -1 to day 28. The median (first and third quartiles, Q1 and Q3) of observed concentration fold changes across all antigens tested is listed, showing a similar and general change in IgG responses to all antigens for each volunteer. To the right, the percentage of antigens within each group, which were considered outliers, is shown, indicating antigens to which increased IgG reactivity was considered antigen-specific, as the increase in IgG reactivity was above 1.5x the interquartile range. Day 28 IgG was from volunteer L1-025 was not analyzed.
(DOCX)

S6 Table Primer sets used for qPCR analysis. (DOCX)

S1 Fig Volunteer antibody response against individual CIDR domains and controls prior to infection (day -1). Heat map showing reactivity of patient plasma samples prior to infection with *Pf*SPZ challenge with different CIDR domain subtypes and control antigens indicated on the left side. Mean fluorescence intensity (MFI) values obtained by luminex assay are shown for each volunteer. Hierarchical clustering with the Euclidian distance using complete linkage method reveals three major groups with different reactivity patterns. First group contains plasma samples mostly from ‘non-controller’ characterized by a high response to AMA1 and low to medium recognition of other antigens. Second group consisting primarily of malaria-naïve samples shows very low reactivity with all antigens tested except tetanus toxin. The third group is formed by plasma samples from ‘clearer’ and ‘controller’ having more antibodies directed against AMA1 and MSP1 as well as CIDR domains.
(PDF)

S2 Fig Mean fluorescence intensities (MFI) of all volunteer sera measured for all antigens by luminex over time. (PDF)

S3 Fig Principal component analysis of ‘non-controller’ and ‘controller’ considering *var* gene expression, luminex data and infection curves of the volunteers. The first two model components summarized 33% and 21% of the data variance, respectively, highlighting high diversity in the underlying data structure. The biplot shows that ‘controller’ and ‘non-controller’ cluster along the first two principal components. They can be separated along the first principal component and ‘controller’ also tend to have lower scores on component 2. However, participant L1-026 (a ‘controller') has the highest score on component 2, which is primarily due to an individual gene expression pattern.
(PDF)

S4 Fig Correlation analysis of samples from two serial parasite generations obtained from volunteers L1-006, L1-010, L1-017 and L1-019. *Ex vivo* samples from two consecutive parasite generations could be obtained from four volunteers. L1-006, L1-017 and L1-019 belong to the group of ‘non-controller’, L1-010 to the ‘controller’ group. Expression data were normalized on total *var* expression in each sample (%) and Spearman’s rank correlation coefficients (*ρ*) indicate a stable *var* gene expression across the parasite replication cycles analyzed. Day of sampling is indicated as day post infection. *Var* genes color-coded consistently with the main figures: red (group A), orange (subfamily *var3*), dark red (subfamily *var1*), purple (group B/A), blue (group B), turquoise (group B/C), green (group C) and yellow (group E).
(PDF)

S5 Fig Quantification of *sbp1* decrease and *var2csa* increase over time in volunteer L1-026 determined by qPCR. RELATEXP values for *sbp1*, *var2csa* and *arginyl-tRNA synthetase* normalized against the housekeeping gene *fructose-bisphosphate aldolase*.
(PDF)

S6 Fig Comparison of Ct values obtained for the normalizer (*sbp1*) and the two housekeeping genes *fructose-bisphosphate aldolase* and *arginyl-tRNA synthetase* in samples from the different volunteer groups. No difference in median expression was observed for all primer sets between the volunteer groups malaria-naïve, ‘non-controller’ and ‘controller’ in regard of RNA content in the samples. Data are shown in box plots extending from the 25^th^ to the 75^th^ percentiles with a line at the median.
(PDF)
