# Correction: Controlled human malaria infection with *Plasmodium falciparum* demonstrates impact of naturally acquired immunity on virulence gene expression

**DOI:** 10.1371/journal.ppat.1014177

**Published:** 2026-04-28

**Authors:** Anna Bachmann, Ellen Bruske, Ralf Krumkamp, Louise Turner, J. Stephan Wichers, Michaela Petter, Jana Held, Michael F. Duffy, B. Kim Lee Sim, Stephen L. Hoffman, Peter G. Kremsner, Bertrand Lell, Thomas Lavstsen, Matthias Frank, Benjamin Mordmüller, Egbert Tannich

S4 Table was uploaded incorrectly. Please see the correct S4 Table below.

## Supporting information

S4 Table Relative expression (RELATEXP) measured by qPCR for all volunteer samples. (XLSX)
