# Supplementary material for: Controlled human malaria infection with Plasmodium falciparum demonstrates impact of naturally acquired immunity on virulence gene expression
Source: PLoS Pathog. 2019 Jul 11;15(7):e1007906. doi: 10.1371/journal.ppat.1007906 (PMC6650087; doi:10.1371/journal.ppat.1007906)
Supplement: S1 Table — (DOCX) [file ppat.1007906.s001.docx]

**Table S1: Overview of volunteer characteristics and parasite counts (parasites/µl) determined by thick blood smear from day 12 to day 28 post infection.**

| volunteer | group | sex | Hb | d12 | | d13 | | d14 | | d15 | | d16 | | d17 | | d18 | | d19 | | d20 | | d21 | | d22 | | d23 | | d24 | | d25 | | d26 | | d27 | | d28 | |  |
| --- | --- | --- | --- | --- | --- | --- | --- | --- | --- | --- | --- | --- | --- | --- | --- | --- | --- | --- | --- | --- | --- | --- | --- | --- | --- | --- | --- | --- | --- | --- | --- | --- | --- | --- | --- | --- | --- | --- |
| L1-001 | malaria-naïve | M | nd | | 0 | | 0 | | 5* | | 0 | | nd | | nd | | nd | | nd | | nd | | nd | | nd | | 0 | | nd | | nd | | nd | | nd | | 0 | |
| L1-002 | ‘clearer’ | M | AS | | 0 | | 0 | | 0 | | 0 | | 0 | | 0 | | 0 | | 0 | | 0 | | 0 | | 0 | | 0 | | 0 | | 0 | | 0 | | 0 | | 0* | |
| L1-003 | ‘controller’ | F | AA | | 0 | | 0 | | 0 | | 0 | | 0 | | 0 | | 0 | | 7 | | 11 | | 10 | | 75 | | 2 | | 52 | | 153 | | 0 | | 7 | | 10* | |
| L1-005 | ‘non-controller’ | F | AS | | 0 | | 0 | | 0 | | 0 | | 0 | | 0 | | 3* | | 19 | | 0 | | 0 | | 0 | | nd | | nd | | 0 | | nd | | nd | | 0 | |
| L1-006 | ‘non-controller’ | M | AA | | 0 | | 10 | | **21** | | **9** | | 5070* | | 0 | | 0 | | 0 | | 0 | | 0 | | nd | | 0 | | nd | | nd | | nd | | nd | | 0 | |
| L1-007 | ‘clearer’ | F | AA | | 0 | | 0 | | 0 | | 0 | | 0 | | 0 | | 0 | | 0 | | 0 | | 0 | | 0 | | 0 | | 0 | | 0 | | 0 | | 0 | | 0* | |
| L1-008 | ‘non-controller’ | M | AA | | 0 | | 0 | | 9 | | 0 | | **1837*** | | 0 | | 0 | | 0 | | 0 | | nd | | nd | | 0 | | nd | | nd | | nd | | nd | | 0 | |
| L1-009 | ‘clearer’ | F | AS | | 0 | | 0 | | 0 | | 0 | | 0 | | 0 | | 0 | | 0 | | 0 | | 0 | | 0 | | 0 | | 0 | | 0 | | 0 | | 0 | | 0* | |
| L1-010 | ‘controller’ | M | AA | | 0 | | 0 | | 0 | | 0 | | 0 | | 0 | | 0 | | 8 | | 0 | | **139** | | 98 | | **117*** | | 0 | | 0 | | 0 | | 0 | | 0 | |
| L1-011 | ‘clearer’ | M | AA | | 0 | | 0 | | 0 | | 0 | | 0 | | 0 | | 0 | | 0 | | 0 | | 0 | | 0 | | 0 | | 0 | | 0 | | 0 | | 0 | | 0* | |
| L1-013 | ‘clearer’ | F | AA | | 0 | | 0 | | 0 | | 0 | | 0 | | 0 | | 0 | | 0 | | 0 | | 0 | | 0 | | 0 | | 0 | | 0 | | 0 | | 0 | | 0* | |
| L1-014 | malaria-naïve | F | nd | | 9* | | 0 | | nd | | 0 | | nd | | 0 | | nd | | 0 | | nd | | nd | | nd | | nd | | nd | | nd | | nd | | nd | | 0 | |
| L1-015 | malaria-naïve | F | nd | | 7* | | 0 | | nd | | 0 | | nd | | 0 | | nd | | 0 | | nd | | nd | | nd | | nd | | nd | | nd | | nd | | nd | | 0 | |
| L1-016 | ‘clearer’ | M | AA | | 0 | | 0 | | 0 | | 0 | | 0 | | 0 | | 0 | | 0 | | 0 | | 0 | | 0 | | 0 | | 0 | | 0 | | 0 | | 0 | | 0* | |
| L1-017 | ‘non-controller’ | M | AS | | 0 | | 0 | | 0 | | 14 | | **38** | | 82 | | 114 | | **675*** | | 96 | | 0 | | 0 | | 0 | | nd | | nd | | 0 | | nd | | 0 | |
| L1-018 | ‘controller’ | M | AS | | 0 | | 0 | | 0 | | 0 | | 0 | | 0 | | 0 | | 0 | | 0 | | 0 | | 0 | | 0 | | 0 | | 10 | | 0 | | 10 | | **52*** | |
| L1-019 | ‘non-controller’ | F | AA | | 0 | | 0 | | 0 | | 0 | | 28 | | **4** | | 318 | | **2423*** | | 28 | | 0 | | 0 | | 0 | | nd | | nd | | 0 | | nd | | 0 | |
| L1-020 | ‘non-controller’ | M | AA | | 0 | | 0 | | 0 | | 0 | | **67*** | | 3 | | 0 | | 0 | | 0 | | nd | | nd | | 0 | | nd | | 0 | | 0 | | nd | | 0 | |
| L1-021 | ‘clearer’ | M | AS | | 0 | | 0 | | 0 | | 0 | | 0 | | 0 | | 0 | | 0 | | 0 | | 0 | | 0 | | 0 | | 0 | | 0 | | 0 | | 0 | | 0* | |
| L1-022 | ‘clearer’ | F | AS | | 0 | | 0 | | 0 | | 0 | | 0 | | 0 | | 0 | | 0 | | 0 | | 0 | | 0 | | 0 | | 0 | | 0 | | 0 | | 0 | | 0* | |
| L1-023 | ‘controller’ | F | AA | | 0 | | 0 | | 0 | | 0 | | 0 | | 0 | | 0 | | 0 | | 0 | | 0 | | 0 | | 0 | | 14 | | **7*** | | 0 | | 0 | | 0 | |
| L1-024 | malaria-naïve | F | nd | | 2* | | 0 | | 0 | | 0 | | 0 | | nd | | nd | | 0 | | nd | | nd | | nd | | nd | | nd | | nd | | nd | | nd | | 0 | |
| L1-025 | malaria-naïve | F | nd | | 4* | | 0 | | 0 | | 0 | | 0 | | nd | | nd | | nd | | 0 | | nd | | nd | | nd | | nd | | nd | | nd | | nd | | 0 | |
| L1-026 | ‘controller’ | M | AS | | 0 | | 0 | | 0 | | 0 | | 0 | | **7** | | 46 | | **59** | | 120 | | **93** | | 0 | | **11** | | 8 | | **19** | | 34 | | 3 | | **26*** | |
| L1-028 | ‘controller’ | M | AS | | 0 | | 0 | | 0 | | 0 | | 0 | | 0 | | 0 | | 0 | | 0 | | 0 | | 0 | | **8** | | 10 | | **81** | | 118 | | 400 | | **164*** | |

* day of treatment

**bold** sample taken for *var* analysis

d day post infection

nd not determined
