# Supplementary material for: Controlled human malaria infection with Plasmodium falciparum demonstrates impact of naturally acquired immunity on virulence gene expression
Source: PLoS Pathog. 2019 Jul 11;15(7):e1007906. doi: 10.1371/journal.ppat.1007906 (PMC6650087; doi:10.1371/journal.ppat.1007906)
Supplement: S2 Table — (DOCX) [file ppat.1007906.s002.docx]

**Table S2: Proteins on luminex plex**

| **Protein ID** | **Protein name** | **Genome/**  **Isolate** | **CIDR Domain class** | **PfEMP1 group** | **Binding phenotype** | **PfEMP1** |
| --- | --- | --- | --- | --- | --- | --- |
| CIDRα1.4_1 | CIDRα1.4_HB3var03_HB3 | HB3 | CIDRα1.4 | A | EPCR | HB3var03 |
| CIDRα1.1_1 | CIDRα1.1_IT4var20_IT4 | FCR3 | CIDRα1.1 | A | EPCR | IT4var20 |
| CIDRα1.4_2 | CIDRα1.4_IT4var07_IT4 | FCR3 | CIDRα1.4 | A | EPCR | IT4var07 |
| CIDRα1.1_2 | CIDRα1.1_igh_var19_IGH | Field isolate | CIDRα1.1 | A | EPCR | igh_var19 |
| CIDRα1.1_3 | CIDRα1.1_raj116_var8_raj116 | Field isolate | CIDRα1.1 | A | EPCR | raj116_var8 |
| CIDRα1.5a_1 | CIDRα1.5a_1965_2_1965 | Field isolate | CIDRα1.5a | A | EPCR | 1965_2 |
| CIDRα1.5a_2 | CIDRα1.5a_GA013_ERS010323 | Sanger | CIDRα1.5a | A | EPCR | GA013 |
| CIDRα1.5a_3 | CIDRα1.5a_GA014_ERS010022 | Sanger | CIDRα1.5a | A | EPCR | GA014 |
| CIDRα1.5b_1 | CIDRα1.5b_1918_5_1918 | Field isolate | CIDRα1.5b | A | unknown | 1918_5 |
| CIDRα1.5b_2 | CIDRα1.5b_1983_13_1983 | Field isolate | CIDRα1.5b | A | unknown | 1983_13 |
| CIDRα1.6a | CIDRα1.6a_HB3var02_HB3 | HB3 | CIDRα1.6a | A | EPCR | HB3var02 |
| CIDRα1.6b_1 | CIDRα1.6b_GA018_ERS010570 | Sanger | CIDRα1.6b | A | EPCR | GA018 |
| CIDRα1.6b_2 | CIDRα1.6b_GA019_ERS010031 | Sanger | CIDRα1.6b | A | EPCR | GA019 |
| CIDRα1.7_1 | CIDRα1.7_1965_8_1965 | Field isolate^1^ | CIDRα1.7 | A | EPCR | 1965_8 |
| CIDRα1.7_2 | CIDRα1.7_1918_3_1918 | Field isolate^1^ | CIDRα1.7 | A | EPCR | 1918_3 |
| CIDRα1.7_3 | CIDRα1.7_GA024_ERS010438 | Sanger | CIDRα1.7 | A | EPCR | GA024 |
| CIDRα1.8a | CIDRα1.8a_GA26_ERS010178 | Sanger | CIDRα1.8a | A | EPCR | GA026 |
| CIDRα1.8b_1 | CIDRα1.8b_GA027_2053 | Field isolate^1^ | CIDRα1.8b | A | EPCR | GA027 |
| CIDRα1.8b_2 | CIDRα1.8bGA029_ERS010532 | Sanger | CIDRα1.8b | A | EPCR | GA029 |
| CIDRα6 | CIDRα6_IT4var12_IT4 | FCR3 | CIDRα6 | B | CD36 | IT4var12 |
| CIDRα5 | CIDRα5_IT4var14_IT4 | FCR3 | CIDRα5 | B | CD36 | IT4var14 |
| CIDRα2.2 | CIDRα2.2_IT4var24_IT4 | FCR3 | CIDRα2.2 | B | CD36 | IT4var24 |
| CIDRα3.3 | CIDRα3.3_IT4var26_IT4 | FCR3 | CIDRα3.3 | B | CD36 | IT4var26 |
| CIDRα2.10 | CIDRα2.10_IT4var30_IT4 | FCR3 | CIDRα2.10 | B | CD36 | IT4var30 |
| CIDRα2.4 | CIDRα2.4_IT4var33_IT4 | FCR3 | CIDRα2.4 | B | CD36 | IT4var33 |
| CIDRα2.9 | CIDRα2.9_IT4var45_IT4 | FCR3 | CIDRα2.9 | B | CD36 | IT4var45 |
| CIDRα2.7 | CIDRα2.7_IT4var61_IT4 | FCR3 | CIDRα2.7 | B | CD36 | IT4var61 |
| CIDRα3.1_1 | CIDRα3.1_Dd2var01_Dd2 | Dd2 | CIDRα3.1 | B | CD36 | Dd2var01 |
| CIDRα3.1_2 | CIDRα3.1_HB3var27_HB3 | HB3 | CIDRα3.1 | B | CD36 | HB3var27 |
| CIDRα3.1_3 | CIDRα3.1_IT4var20_IT4 | FCR3 | CIDRα3.1 | B | CD36 | IT4var20 |
| CIDRα3.5 | CIDRα3.5_IT4var07_IT4 | FCR3 | CIDRα3.5 | B | CD36 | IT4var07 |
| CIDRδ1_1 | CIDRδ1_HB3var05_HB3 | HB3 | CIDRδ1 | A | unknown | HB3var05 |
| CIDRδ1_2 | CIDRδ1_HB3var35_HB3 | HB3 | CIDRδ1 | A | unknown | HB3var35 |
| CIDRδ1_3 | CIDRδ1__IT4var02_IT4 | FCR3 | CIDRδ1 | A | unknown | IT4var02 |
| CIDRγ3 | CIDRγ3_IT4var08_IT4 | FCR3 | CIDRγ3 | A | unknown | IT4var08 |
| AMA1 |  |  |  |  |  |  |
| MSP1 |  |  |  |  |  |  |
| CSP |  |  |  |  |  |  |
| VAR2 | VAR2CSA (minimal binding region) | 3D7 |  | E | CSA | PFL00030c |
| TetTox |  |  |  |  |  |  |
| BSA |  |  |  |  |  |  |
