# Supplementary material for: Controlled human malaria infection with Plasmodium falciparum demonstrates impact of naturally acquired immunity on virulence gene expression
Source: PLoS Pathog. 2019 Jul 11;15(7):e1007906. doi: 10.1371/journal.ppat.1007906 (PMC6650087; doi:10.1371/journal.ppat.1007906)
Supplement: S3 Table — For each donor at day -1 and 28, the proportion (%) of CIDRα2–6 (n = 12), CIDRα1 (n = 19) and CIDRδ/γ (n = 4) domains recognized at IgG levels above the mean plus two standard deviations of the IgG levels of the naïve donors at day -1, is listed. nd = not determined. * day 19 (DOCX) [file ppat.1007906.s003.docx]

|  |  | CIDRα2-6  CD36-binding | | CIDRα1  EPCR-binding | | CIDRδ/γ  other group A | |
| --- | --- | --- | --- | --- | --- | --- | --- |
| volunteer ID | **group** | **day -1** | **day 28** | **day -1** | **day 28** | **day -1** | **day 28** |
| L1-002 | ’clearer’ | 0 | 8,3 | 15,8 | 26,3 | 0 | 0 |
| L1-007 | ’clearer’ | 41,7 | 50 | 63,2 | 89,5 | 25 | 50 |
| L1-009 | ’clearer’ | 16,7 | 25 | 52,6 | 68,4 | 50 | 75 |
| L1-011 | ’clearer’ | 25 | 16,7 | 31,6 | 36,8 | 25 | 25 |
| L1-013 | ’clearer’ | 0 | 8,3 | 0 | 15,8 | 0 | 0 |
| L1-016 | ’clearer’ | 8,3 | 16,7 | 52,6 | 73,7 | 75 | 75 |
| L1-021 | ’clearer’ | 100 | 83, 3 | 94,7 | 84,2 | 100 | 100 |
| L1-022 | ’clearer’ | 66, 7 | 25 | 78,9 | 73,6 | 100 | 100 |
| L1-003 | ’controller’ | 25 | 25 | 47,4 | 47,4 | 25 | 25 |
| L1-010 | ’controller’ | 16, 7 | 83, 3 | 57,9 | 100 | 50 | 100 |
| L1-018 | ’controller | 50 | 66, 7 | 63,2 | 73,7 | 100 | 100 |
| L1-026 | ’controller’ | 25 | 50 | 63,2 | 78,9* | 0 | 25 |
| L1-028 | ’controller’ | 8,3 | 41, 7 | 5,3 | 26,3 | 25 | 50 |
| L1-001 | malaria-naïve | 0 | 0 | 0 | 5,3 | 0 | 0 |
| L1-014 | malaria-naïve | 0 | 8,3 | 0 | 21,1 | 0 | 0 |
| L1-015 | malaria-naïve | 0 | 25 | 0 | 10,5 | 0 | 25 |
| L1-024 | malaria-naïve | 0 | 0 | 0 | 0 | 0 | 25 |
| L1-025 | malaria-naïve | 0 | **nd** | 0 | **nd** | 0 | **nd** |
| L1-005 | ’non-controller’ | 8,3 | 33,3 | 0 | 10,5 | 0 | 100 |
| L1-006 | ’non-controller’ | 0 | 16,7 | 5,3 | 26,3 | 0 | 25 |
| L1-008 | ’non-controller’ | 0 | 8,3 | 0 | 5,3 | 0 | 0 |
| L1-017 | ’non-controller’ | 8,3 | 33,3 | 15,8 | 68,4 | 25 | 75 |
| L1-019 | ’non-controller’ | 0 | 33, 3 | 5,3 | 42,1 | 25 | 25 |

**Table S3: Breadth of IgG recognition of CIDR domains for each donor.** For each donor at day -1 and 28, the proportion (%) of CIDRα2-6 (n=12), CIDRα1 (n=19) and CIDRδ/γ (n=4) domains recognized at IgG levels above the mean plus two standard deviations of the IgG levels of the naïve donors at day -1, is listed. nd = not determined.* day 19
