# Supplementary material for: Controlled human malaria infection with Plasmodium falciparum demonstrates impact of naturally acquired immunity on virulence gene expression
Source: PLoS Pathog. 2019 Jul 11;15(7):e1007906. doi: 10.1371/journal.ppat.1007906 (PMC6650087; doi:10.1371/journal.ppat.1007906)
Supplement: S5 Table — The median (first and third quartiles, Q1 and Q3) of observed concentration fold changes across all antigens tested is listed, showing a similar and general change in IgG responses to all antigens for each volunteer. To the right, the percentage of antigens within each group, which were considered outliers, is shown, indicating antigens to which increased IgG reactivity was considered antigen-specific, as the increase in IgG reactivity was above 1.5x the interquartile range. Day 28 IgG was from volunteer L1-025 was not analyzed. (DOCX) [file ppat.1007906.s005.docx]

**Table S5. Change in IgG levels from day -1 to day 28.** The median (first and third quartiles, Q1 and Q3) of observed concentration fold changes across all antigens tested is listed, showing a similar and general change in IgG responses to all antigens for each volunteer. To the right, the percentage of antigens within each group, which were considered outliers, is shown, indicating antigens to which increased IgG reactivity was considered antigen-specific, as the increase in IgG reactivity was above 1.5x the interquartile range. Day 28 IgG was from volunteer L1-025 was not analyzed.

|  |  | IgG reactivity fold change day-1 to 28 | | sero-conversion (% of antigens) | | | | | | | |
| --- | --- | --- | --- | --- | --- | --- | --- | --- | --- | --- | --- |
| volunteer ID | **group** | **median** | **Q1 – Q3** | **CIDRα1 (EPCR)** | **CIDRα2-6 (CD36)** | **AMA1** | **MSP1** | **CSP** | **VAR2** | **TetTox** | **BSA** |
| L1-002 | ’clearer’ | 2.0 | 1.9 – 2.1 | 0 | 0 | 0 | 0 | 0 | 0 | 0 | 0 |
| L1-007 | ’clearer’ | 2.3 | 2.1 – 2.4 | 0 | 0 | 0 | 0 | 0 | 0 | 0 | 0 |
| L1-009 | ’clearer’ | 1.7 | 1.6 – 1.8 | 0 | 0 | 0 | 0 | 0 | 0 | 0 | 0 |
| L1-011 | ’clearer’ | 1.0 | 0.8 – 1.0 | 0 | 0 | 0 | 0 | 0 | 0 | 0 | 0 |
| L1-013 | ’clearer’ | 4.6 | 4.2 – 5.5 | 0 | 8.3 | 0 | 0 | 0 | 0 | 0 | 0 |
| L1-016 | ’clearer’ | 1.3 | 1.3 – 1.4 | 0 | 0 | 0 | 0 | 0 | 0 | 0 | 0 |
| L1-021 | ’clearer’ | 0.8 | 0.8 – 0.9 | 0 | 0 | 0 | 0 | 0 | 0 | 0 | 0 |
| L1-022 | ’clearer’ | 0.7 | 0.5 – 1.0 | 0 | 0 | 0 | 0 | 0 | 0 | 0 | 0 |
| L1-003 | ’controller’ | 1.1 | 1.0 – 1.3 | 0 | 0 | 0 | 0 | 0 | 0 | 0 | 0 |
| L1-010 | ’controller’ | 6.0 | 5.2 – 7.4 | 5.9 | 0 | 0 | 0 | 0 | 100 | 0 | 0 |
| L1-018 | ’controller’ | 1.2 | 1.1 – 1.3 | 0 | 0 | 0 | 0 | 0 | 0 | 0 | 0 |
| L1-026 | ’controller’ | 1.3 | 1.1 – 1.5 | 0 | 0 | 100 | 100 | 0 | 0 | 0 | 0 |
| L1-028 | ’controller’ | 2.0 | 1.3 – 5.1 | 0 | 0 | 0 | 0 | 0 | 0 | 0 | 0 |
| L1-001 | malaria-naïve | 0.9 | 0.9 – 1.0 | 0 | 0 | 100 | 100 | 0 | 0 | 0 | 0 |
| L1-014 | malaria-naïve | 9.6 | 8.3 – 10.2 | 0 | 0 | 100 | 100 | 100 | 0 | 0 | 0 |
| L1-015 | malaria-naïve | 1.9 | 1.7 – 2.4 | 5.9 | 0 | 100 | 100 | 0 | 0 | 0 | 0 |
| L1-024 | malaria-naïve | 2.0 | 1.8 – 2.3 | 5.9 | 0 | 0 | 100 | 0 | 0 | 0 | 0 |
| L1-005 | ’non-controller’ | 2.2 | 1.8 – 3.3 | 0 | 33.3 | 0 | 100 | 100 | 0 | 0 | 0 |
| L1-006 | ’non-controller’ | 1.8 | 1.6 – 2.1 | 0 | 0 | 100 | 0 | 0 | 0 | 0 | 0 |
| L1-008 | ’non-controller’ | 9.3 | 7.5 – 12.4 | 11.8 | 8.3 | 100 | 0 | 0 | 0 | 0 | 0 |
| L1-017 | ’non-controller’ | 2.7 | 1.8 – 3.5 | 0 | 8.3 | 0 | 0 | 100 | 0 | 0 | 0 |
| L1-019 | ’non-controller’ | 2.6 | 1.8 – 7.7 | 23.5 | 0 | 0 | 0 | 0 | 0 | 0 | 0 |
| L1-020 | ’non-controller’ | 0.7 | 0.6 – 0.8 | 0 | 0 | 0 | 0 | 100 | 0 | 0 | 0 |
