# Supplementary material for: Controlled human malaria infection with Plasmodium falciparum demonstrates impact of naturally acquired immunity on virulence gene expression
Source: PLoS Pathog. 2019 Jul 11;15(7):e1007906. doi: 10.1371/journal.ppat.1007906 (PMC6650087; doi:10.1371/journal.ppat.1007906)
Supplement: S6 Table — (DOCX) [file ppat.1007906.s006.docx]

**Table S6: Primer sets used for qPCR analysis.**

| **Primer ID** | **New gene ID** | **Gene ID** | **Forward primer (5’→3’)** | **Reverse primer (5’→3’)** | **Efficiency** |
| --- | --- | --- | --- | --- | --- |
| AS1^1^ | PF3D7_0100100 | PFA0005w | TGCGCTGATAACTCACAACA | AGGGGTTCATCGTCATCTTC | 2,025 |
| AS4^1^ | PF3D7_0421104 | PF10_0001 | GACGAGGAGTCGGAAAAGAC | TGGACAGGCTTGTTTGAGAG | 1,983 |
| AS6^1^ | PF3D7_1100100 | PF11_0007 | GAGGCTTATGGGAAACCAGA | AGGCAGTCTTTGGCATCTTT | 1,975 |
| AS7^1^ | PF3D7_1100200 | PF11_0008 | GACGGCTACCACAGAGACAA | CGTCATCATCGTCTTCGTTT | 1,949 |
| AB2^8^ | PF3D7_1240300 | PFL1950w | ACGCAGAAGTACAAAGAGATGC | ATCCGGTGATGTCGTTCCTT | 1,948 |
| MF2^5^ | PF3D7_1255200 | PFL2665c | GCGAGGTCTTCTCGTTCTTG | ATGACGAAGAAGCAGCAGGT | 1,951 |
| AS18^1^ | PF3D7_1200100 | PFL0005w | CGGAGGAGGAAAAACAAGAG | TGCCGTATTTGAGACCACAT | 1,947 |
| AS20^1^ | PF3D7_1300300 | PF13_0003 | CACAGGTATGGGAAGCAATG | CCATACAGCCGTGACTGTTC | 2,040 |
| AS23^1^ | PF3D7_0324900, PF3D7_0300100 | PFC1120c, PFC0005w | CAATCTGCGGCAATAGAGAC | CCACTGTTGAGGGGTTTTCT | 1,937 |
| AS26^1^ | PF3D7_0412400 | PFD0615c | ACCGCCCCATCTAGTGATAG | CACTTGGTGATGTGGTGTCA | 2,016 |
| AS27^1^ | PF3D7_0412700 | PFD0625c | TAAAAGACGCCAACAGATGC | TCATCGTCTTCGTCTTCGTC | 2,018 |
| AS30^1^ | PF3D7_0400100 | PFD0005w | GACGACGATGAAGACGAAGA | AGATCTCCGCATTTCCAATC | 2,007 |
| AS34^1^ | PF3D7_0421300 | PFD1015c | TGCAACGAAACATTAGCACA | AGCAGGGGATGATGCTTTAC | 1,901 |
| AS35^1^ | PF3D7_0425800 | PFD1235w | AAACACGTTGAATGGCGATA | GACGCCGAGGAGGTAAATAG | 1,905 |
| AS36^1^ | PF3D7_0426000 | PFD1245c | TGACGACTCCTCAGACGAAG | CTCCACTGACGGATCTGTTG | 1,863 |
| AS37*^1^ | PF3D7_0533100 | PFE1640w | AAGAAAGTGCCACAACATGC | GTTCGTACGCCTGTCGTTTA | 1,975 |
| AB3^8^ | PF3D7_0500100 | PFE0005w | GAGTGGTGGTAACACGGAGA | ATCTTGTGACGCAGTTTGGG | 1,958 |
| AS39^1^ | PF3D7_0617400 | MAL6P1.252 | ATTTGTCGCACATGAAGGAA | AACTTCGTGCCAATGCTGTA | 1,883 |
| MF3^5^ | PF3D7_0800300 | PF08_0140 | GGAGGAGGAAGAGGAAAACG | CCACCTCCTCTTGTTGTGGT | 1,961 |
| AS43^1^ | PF3D7_0800100 | PF08_0142 | GTCGTGGAAAAACGAAAGGT | TATCTATCCAGGGCCCAAAG | 1,889 |
| AS44^1^ | PF3D7_0632500 | MAL6P1.4 | ATGTGTGCGAGAAGGTGAAG | TGCCTTCTAGGTGGCATACA | 1,911 |
| AS45^1^ | PF3D7_0711700 | PF07_0048 | CAATTTTTCCGACGCTTGTA | CACATATAGCGCCGTCCTTA | 1,886 |
| MF4^5^ | PF3D7_0712300 | MAL7P1.50 | GGTGGAGGTAGTCCACAGGA | CAGCTATTTCCCCACCAGAA | 1,957 |
| AB1^8^ | PF3D7_0712000 | PF07_0049 | ATGAATTTGGGCAAAAAGTGTACG | TCATTCCAAATTGGTGCTAGTGA | 1,921 |
| AS50^1^ | PF3D7_0712900 | MAL7P1.56 | CACACATGTCCACCACAAGA | ACCCTTCTGTGGTGTCTTCC | 1,933 |
| AS51^1^ | PF3D7_0712800 | MAL7P1.55 | ACGTGGTGGAGACGTAAACA | CCTTTGTTGTTGCCACTTTG | 1,901 |
| AB4^8^ | PF3D7_0712600 | PF07_0051 | TGCACGACCAAATGAAAAAGGA | ATCGGTGGCACCTGTTTCTC | 1,902 |
| AS55^1^ | PF3D7_0808700 | PF08_0106 | TTTGTCCGGAAGACGATACA | ATCTGGGGCAGAATTACCAC | 1,835 |
| AS56^1^ | PF3D7_0900100 | PFI0005w | TGCAAACCACCAGAAGAAAG | GTTCTCCGTGTTGTCCTCCT | 1,977 |
| AS91^1^ | PF3D7_1300100 | PF13_0001 | ACAAAGGAACGTCCATCTCC | GCCAATACTCCACATGATCG | 1,916 |
| AS92^1^ | PF3D7_0809100 | PF08_0103 | TGCAAGGGTGCTAATGGTAA | CCTGCATTTTGACATTCGTC | 1,841 |
| AS93^1^ | PF3D7_0632800 | MAL6P1.1 | GACAAATACGGCGACTACGA | TGTTTCACCCCATTCTTCAA | 1,975 |
| AS95^1^ | PF3D7_0420700 | PFD0995c | TCACAACCTGACCCCCTACT | TCTTCGTCGTTGTCATCCTC | 2,000 |
| AS96^1^ | PF3D7_0937600 | PFI1820w | TGACCAAGACGAAGTATGGAA | TTGATCTCTGTTCGCTGTCC | 1,991 |
| TL1^4^ | PF3D7_0937800 | PFI1830c | ACAACAATTTCGCAAGCAAG | TTCCTCTGCCTCCTCTTCAT | 2,084 |
| WT1^2^ | PF3D7_0600400 | PFF0020c | GCACATTATCAAACGCCC | AACCAGCTGCCTTGTGCAA | 1,994 |
| RD1^3^ | PF3D7_0808600 | PF08_0107 | CCTAAAAAGGACGCAGAAGG | CCAGCAACACTACCACCAGT | 2,001 |
| MDa500/501^6^ | PF3D7_0700100 | MAL8P1.220 | GTCCTCTATGTGGAGTGAAAAAGAA | AGTACCGTTATCTGGGTTTATAGGC | 2,011 |
| MDa498/499^6^ | PF3D7_0833500 | MAL7P1.212 | AATCAGAAAAGTGTAATTGCAGGAG | TTTACTATCATCACTGACACGCATT | 1,972 |
| AB8 | PF3D7_1240600 | PFL1960w | ACAAATAGTGATCCTGTAATGAACC | TGTTTGTATCCCACTTTTCGC | 2,069 |
| AB9 | PF3D7_1373500 | PF13_0364 | CAAGGAGGTAGCGGTGATCC | TAGCCTCACCATGCACTTCG | 1,999 |
| AB10 | PF3D7_0223500 | PFB1055c | GTGGTAAGGGCGGTGATCC | AGTTTCACTTTTCACTTGCTCGT | 2,067 |
| AB15 | PF3D7_1219300 | PFL0935c | TAATGTCGCCAAACCTGCAC | TCCACTTTATTGTTTGTATCCCACT | 1,984 |
| AB16 | PF3D7_0421100 | PFD1005c | GTGGTAAAGACGGAGCCACT | CCTTCACGTTGTCGTCCACT | 2,025 |
| AB17 | PF3D7_0600200 | MAL6P1.316 | GGAAGGAAATTTGGCAAGCTCA | TATTTCCGCACGGATGCCTT | 1,944 |
| AB18 | PF3D7_1240400 | PFL1955w | TGATGGCACAATCCCACCAG | AACGTGTCAATCATCGTGGT | 1,944 |
| AB19 | PF3D7_1240900 | PFL1970w | ACAGAAATGGTGGAAGAGGTGA | GCCGGAAGTGTAGTAGGATCG | 1,936 |
| AB20 | PF3D7_0412900 | PFD0630c | AGGGTGTGGATGACCGAAAC | TCCCATTTTCTTCGCCGTTC | 2,016 |
| AB21 | PF3D7_0413100 | PFD0635c | GGGTGTGGATGACCGAAACT | TCCTTTTCAGACGTATTTGCACC | 1,985 |
| AB23 | PF3D7_0420900 | PFD1000c | TGGTGATAAGGACGGTGCCA | CGTCCTTCACGTTGTCGTCC | 1,936 |
| AB24 | PF3D7_0200100 | PFB0010w | TCCACCAACTAGTGACATACCT | GAAACATCAGTATTCAACGTTTTGT | 1,930 |
| AB25 | PF3D7_0400400 | PFD0020c | ACAAGTCAATTGAGAGGCGA | TCGCATGAATTTGCAGGACC | 1,940 |
| AB26 | PF3D7_0733000 | PF07_0139 | TGGTAAACAAGTGTTGAATACGGA | TCATCCACTTGGTTGGGGTT | 1,990 |
| AB27 | PF3D7_1041300 | PF10_0406 | GGATACAACTGCCAAACATGC | TCTGACAAACGTCCATGCAA | 1,987 |
| AB46 | PF3D7_0115700 | PFA0765c | TGGTGACTGGTAGTGGTGGT | TTTCGTGCACGTCTTTCCCA | 1,920 |
| AB47 | PF3D7_1100100 | PF11_0007 | ATGGAGCCGCATGGAGG | TGGTCGTGCACTATTTTCCCT | 1,982 |
| AB48 | PF3D7_1150400 | PF11_0521 | AGCGACTCCGGATCCAATTT | ACATCTTTTGTTGCTTTCGCT | 1,912 |
| AB49 | PF3D7_1200400 | PFL0020w | ACGCGTGCGTCTGACTATAA | CCTACCACGCGTTAGAGCTT | 1,896 |
| AB50 | PF3D7_1200600 | PFL0030c | TGGAAGTGGAGGTGATGGAT | GGAGGTGGTATTCTATCACAAGGA | 2,056 |
| AB51 | PF3D7_0617400 | MAL6P1.252 | CTGACGAACCCGATGAGGAG | TCCTCTTGTTTTGGTGGTGCT | 1,965 |
| AB52 | PF3D7_0800200 | PF08_0141 | ACCTGTGGATACAAGCGATGT | TCTGCATCTTCTTTCACTCGGT | 1,947 |
| AB54 | PF3D7_0712400 | PF07_0050 | TGGGAAGCAAAATTTGTTGGTGT | TGGGTCATCTTTTCCTGTCGT | 2,000 |
| AB56 | PF3D7_0100300 | PFA0015c | ACCCCTACACGTCACCTAAA | ACCATCACCCTTACTTGGTGT | 2,061 |
| AS61b **^1^ | PF3D7_1444800 | PF14_0425 | TGTACCACCAGCCTTACCAG | TTCCTTGCCATGTGTTCAAT | 1,974 |
| MD93/ 94***^7^ | PF3D7_0501300 | PFE0065w | TTAGCCGACGAACCAACACA | TTCGGTTGTCTCTGGTACTGCA | 1,951 |
| AB41** | PF3D7_1218600 | PFL0900c | TTCAAAACACGAAGTGGAACAAC | AATTCTCTGCAGCAAGTCGC | 1,964 |

1: Salanti *et al.* (2003) Mol Microbiol * pseudogene

2: Tham *et al.* (2007) Int J Parasitol ** housekeeping genes

3: Dzikowski *et al.* (2006) Plos Path *** sbp1 (ring stage control, normalizer)

4: Lavstsen *et al.* (2005) Mal J

5: Frank *et al.* (2007) Mol Microbiol

6: Dahlbaeck *et al.* (2007) Mal J

7: Petter *et al.* (2011) Plos Path

8: Bachmann *et al.* (2016) Plos Path
