# Supplementary figures and images for: Controlled human malaria infection with Plasmodium falciparum demonstrates impact of naturally acquired immunity on virulence gene expression
Source: PLoS Pathog. 2019 Jul 11;15(7):e1007906. doi: 10.1371/journal.ppat.1007906 (PMC6650087; doi:10.1371/journal.ppat.1007906)

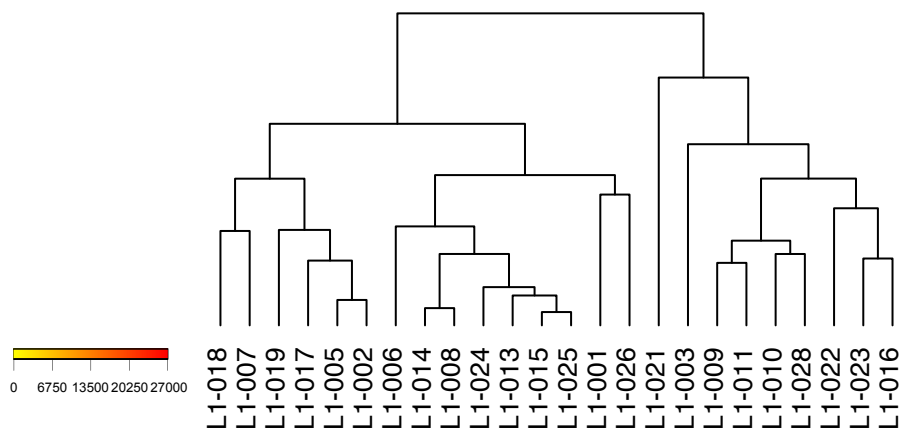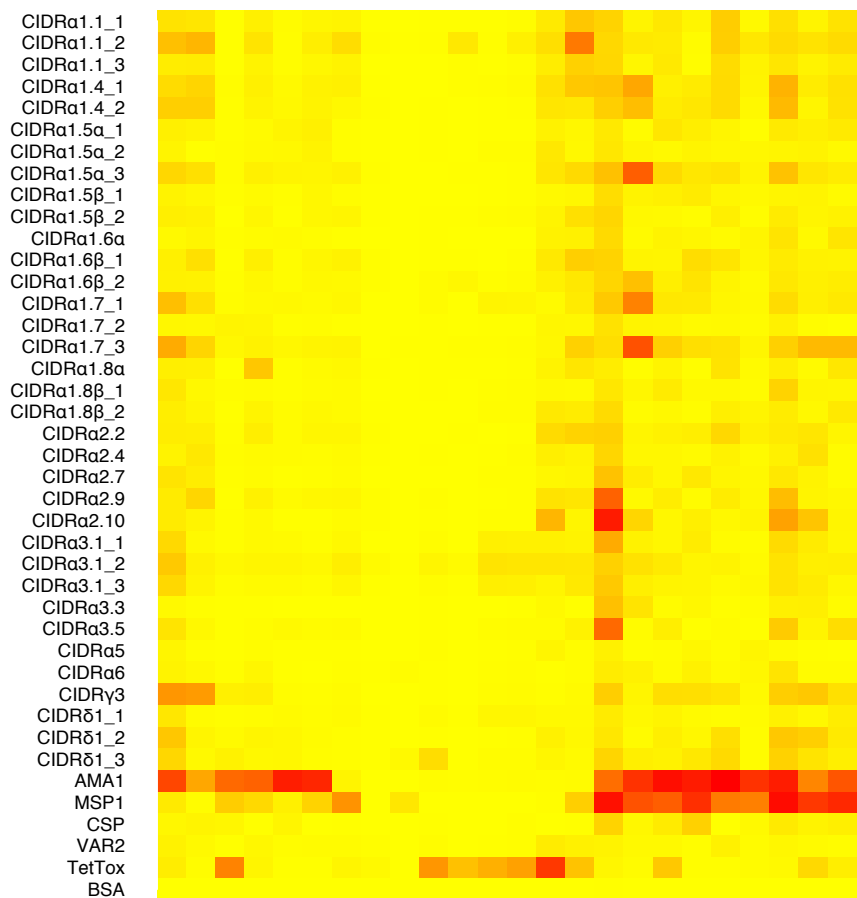

Supplement: S1 Fig — Heat map showing reactivity of patient plasma samples prior to infection with PfSPZ challenge with different CIDR domain subtypes and control antigens indicated on the left side. Mean fluorescence intensity (MFI) values obtained by luminex assay are shown for each volunteer. Hierarchical clustering with the Euclidian distance using complete linkage method reveals three major groups with different reactivity patterns. First group contains plasma samples mostly from ‘non-controller’ characterized by a high response to AMA1 and low to medium recognition of other antigens. Second group consisting primarily of malaria-naïve samples shows very low reactivity with all antigens tested except tetanus toxin. The third group is formed by plasma samples from ‘clearer’ and ‘controller’ having more antibodies directed against AMA1 and MSP1 as well as CIDR domains. (PDF) [file ppat.1007906.s007.pdf]

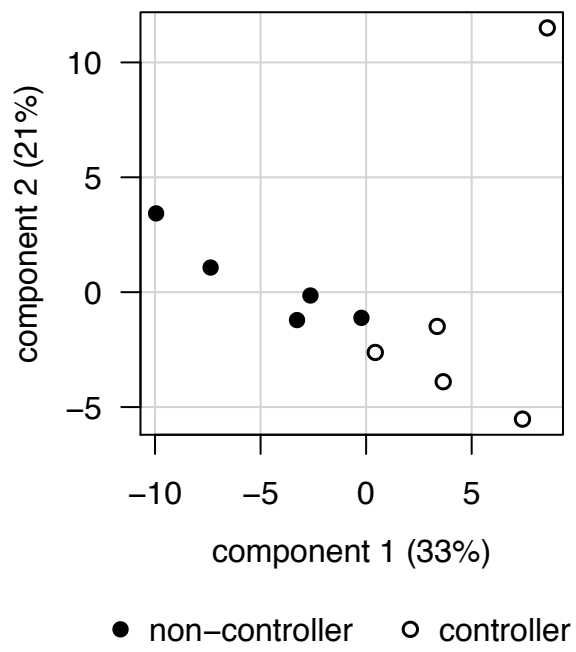

Supplement: S3 Fig — The first two model components summarized 33% and 21% of the data variance, respectively, highlighting high diversity in the underlying data structure. The biplot shows that ‘controller’ and ‘non-controller’ cluster along the first two principal components. They can be separated along the first principal component and ‘controller’ also tend to have lower scores on component 2. However, participant L1-026 (a ‘controller') has the highest score on component 2, which is primarily due to an individual gene expression pattern. (PDF) [file ppat.1007906.s009.pdf]

**L1-006**

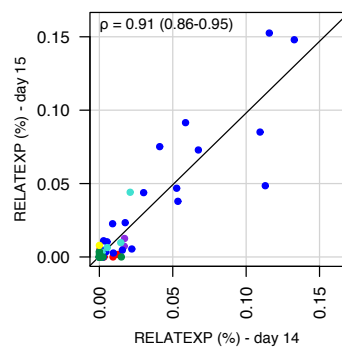

**L1-010**

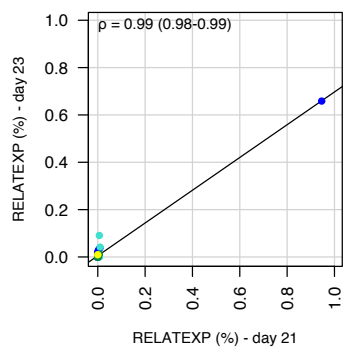

**L1-017**

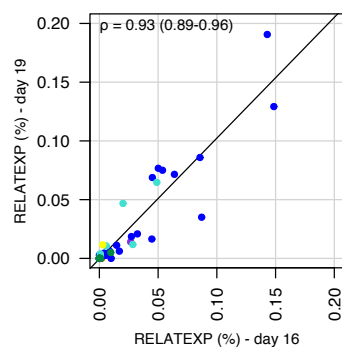

**L1-019**

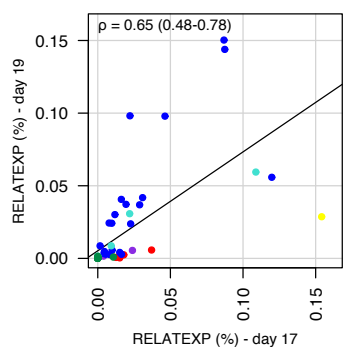

Supplement: S4 Fig — Ex vivo samples from two consecutive parasite generations could be obtained from four volunteers. L1-006, L1-017 and L1-019 belong to the group of ‘non-controller’, L1-010 to the ‘controller’ group. Expression data were normalized on total var expression in each sample (%) and Spearman’s rank correlation coefficients (ρ) indicate a stable var gene expression across the parasite replication cycles analyzed. Day of sampling is indicated as day post infection. Var genes color-coded consistently with the main figures: red (group A), orange (subfamily var3), dark red (subfamily var1), purple (group B/A), blue (group B), turquoise (group B/C), green (group C) and yellow (group E). (PDF) [file ppat.1007906.s010.pdf]

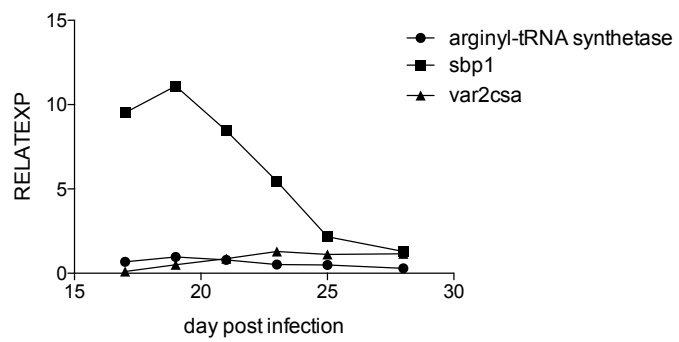

Supplement: S5 Fig — RELATEXP values for sbp1, var2csa and arginyl-tRNA synthetase normalized against the housekeeping gene fructose-bisphosphate aldolase. (PDF) [file ppat.1007906.s011.pdf]

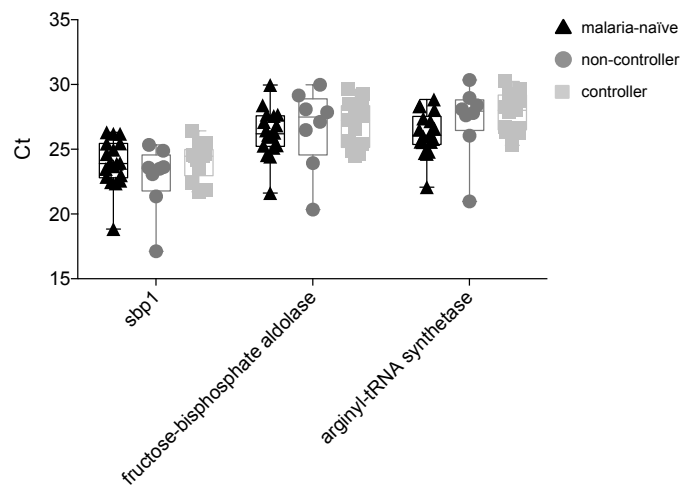

Supplement: S6 Fig — No difference in median expression was observed for all primer sets between the volunteer groups malaria-naïve, ‘non-controller’ and ‘controller’ in regard of RNA content in the samples. Data are shown in box plots extending from the 25th to the 75th percentiles with a line at the median. (PDF) [file ppat.1007906.s012.pdf]
