# Supplementary material for: Controlled human malaria infection with Plasmodium falciparum demonstrates impact of naturally acquired immunity on virulence gene expression
Source: PLoS Pathog. 2019 Jul 11;15(7):e1007906. doi: 10.1371/journal.ppat.1007906 (PMC6650087; doi:10.1371/journal.ppat.1007906)
Supplement: S2 Fig — (PDF) [file ppat.1007906.s008.pdf]

CIDRα1.1_1

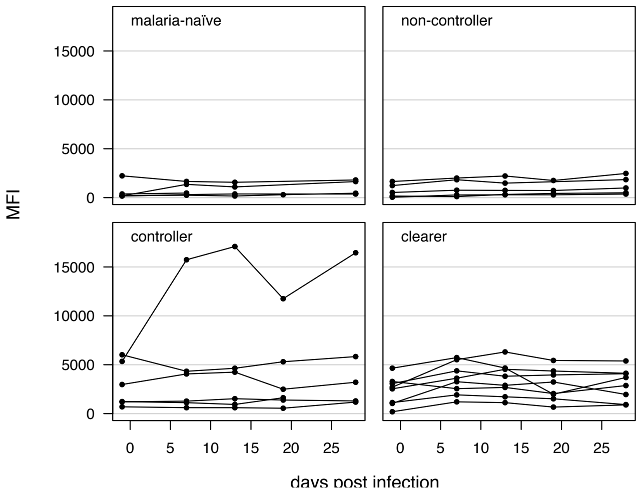

CIDRα1.1_2

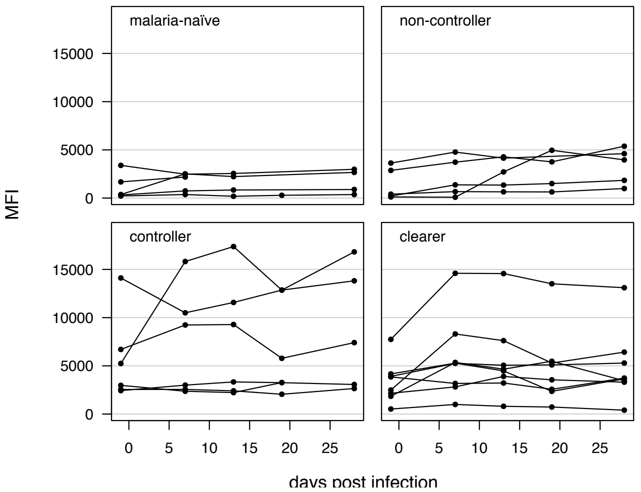

CIDRα1.1_3

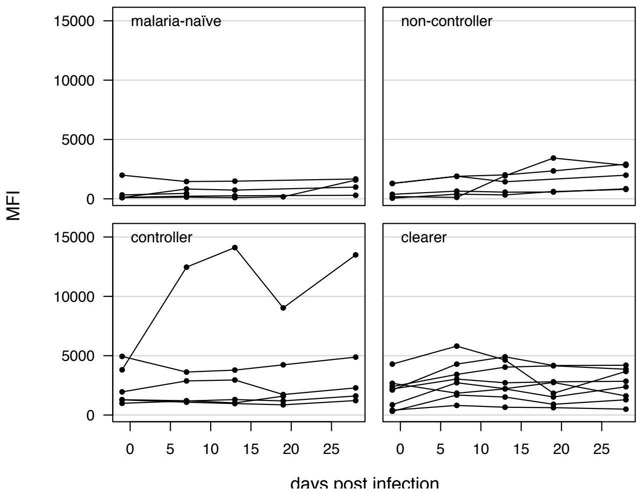

CIDRα1.4_1

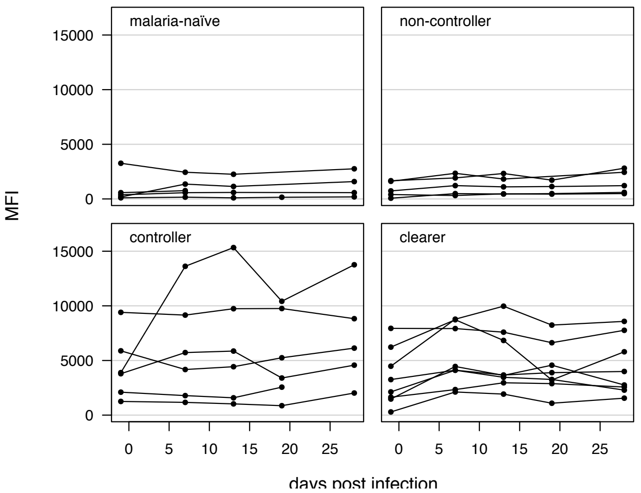

CIDRα1.4_2

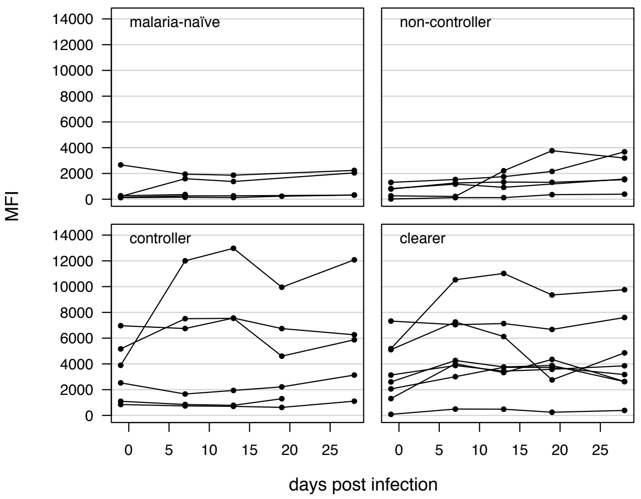

CIDRα1.5a_1

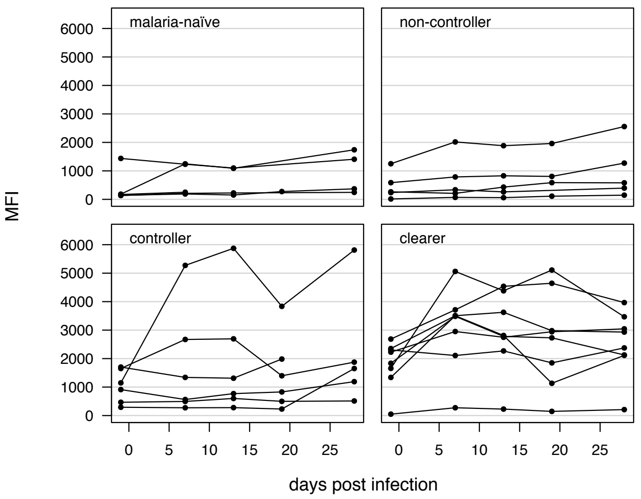

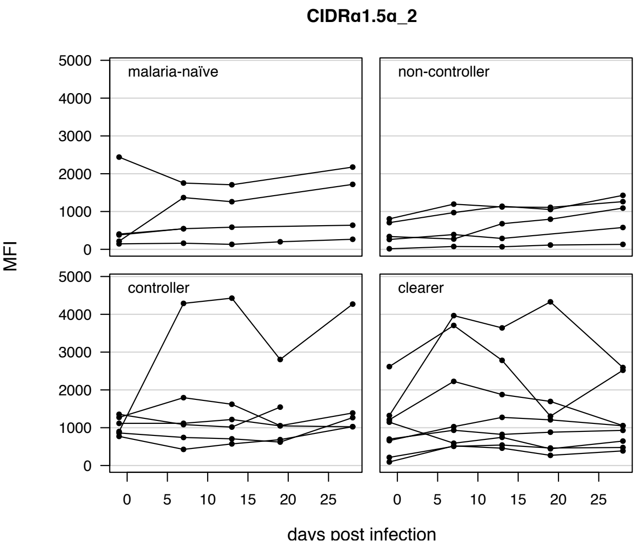
CIDRα1.5α_2
malaria-naïve
non-controller
controller
clearer
MFI
days post infection

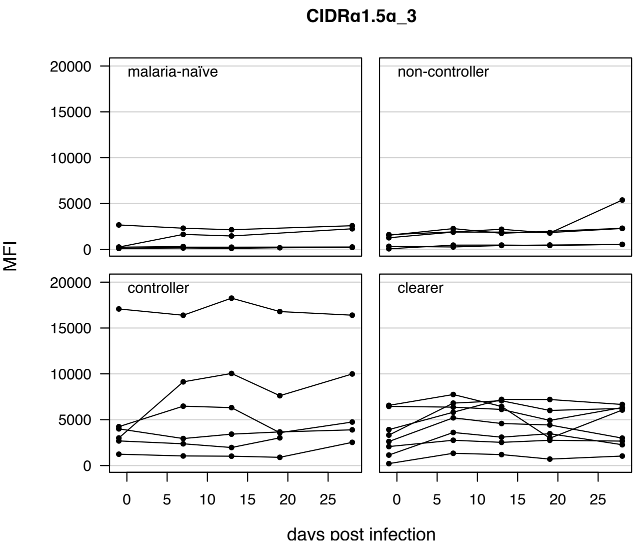
CIDRα1.5α_3
malaria-naïve
non-controller
controller
clearer
MFI
days post infection

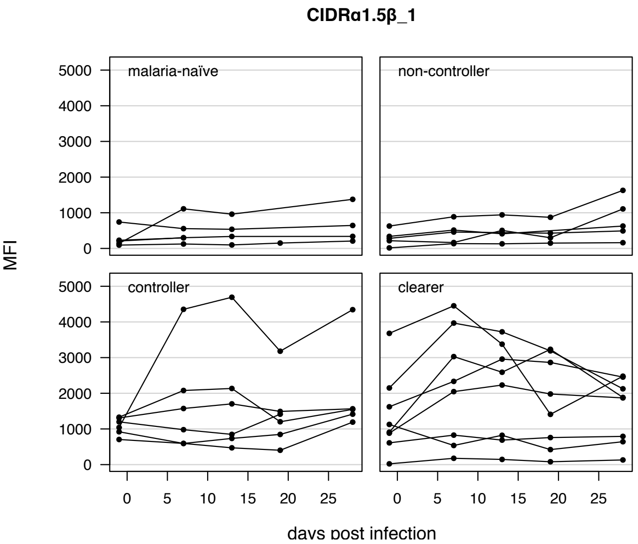
CIDRα1.5β_1
malaria-naïve
non-controller
controller
clearer
MFI
days post infection

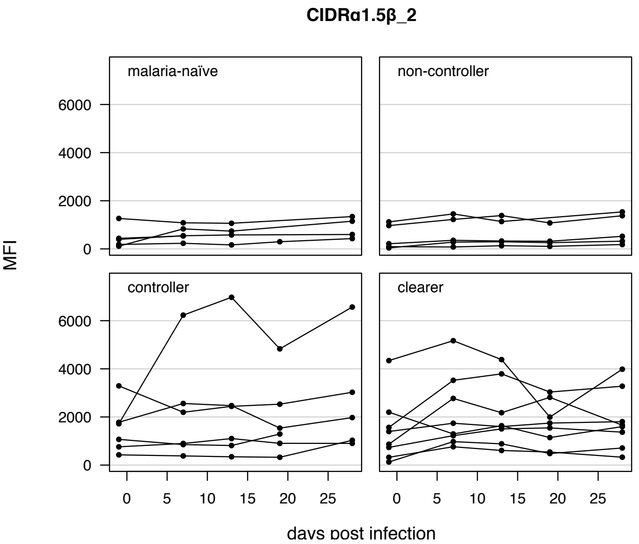
CIDRα1.5β_2
malaria-naïve
non-controller
controller
clearer
MFI
days post infection

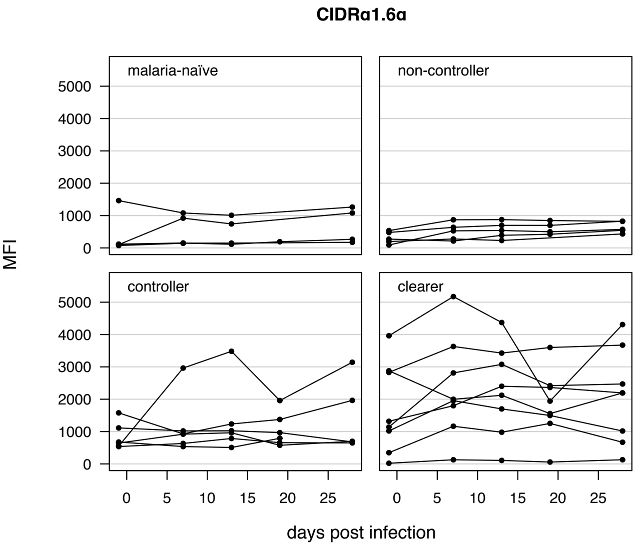
CIDRα1.6α
malaria-naïve
non-controller
controller
clearer
MFI
days post infection

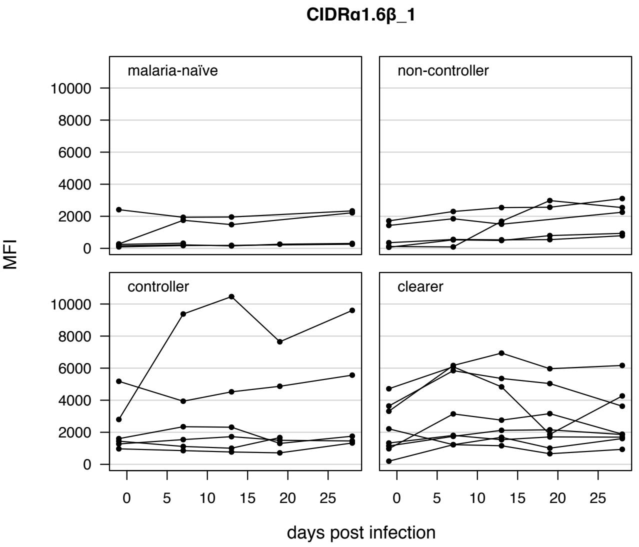
CIDRα1.6β_1
malaria-naïve
non-controller
controller
clearer
MFI
days post infection

CIDRα1.6β_2

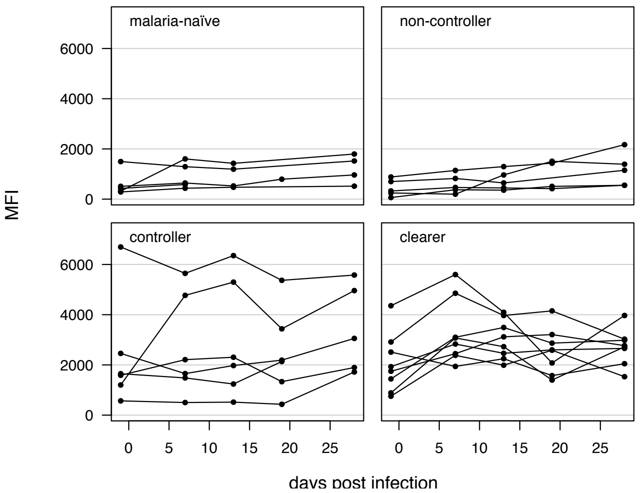

CIDRα1.7_1

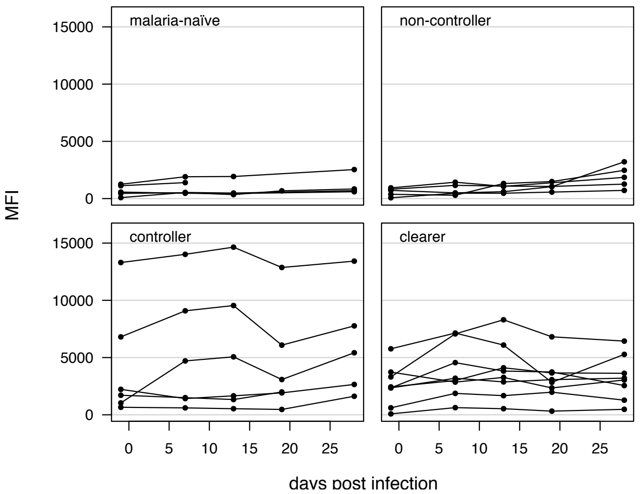

CIDRα1.7_2

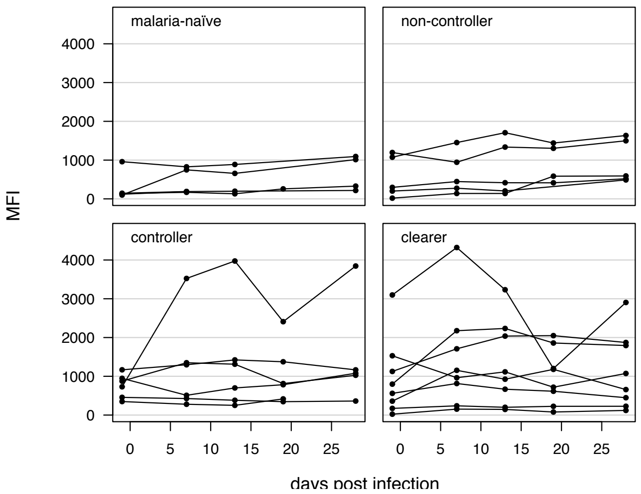

CIDRα1.7_3

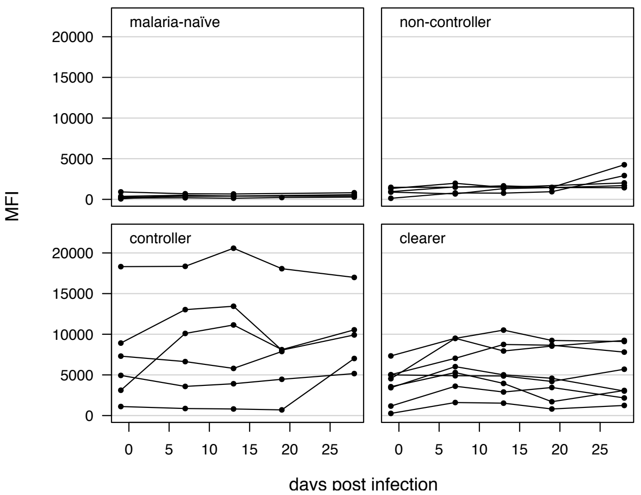

CIDRα1.8α

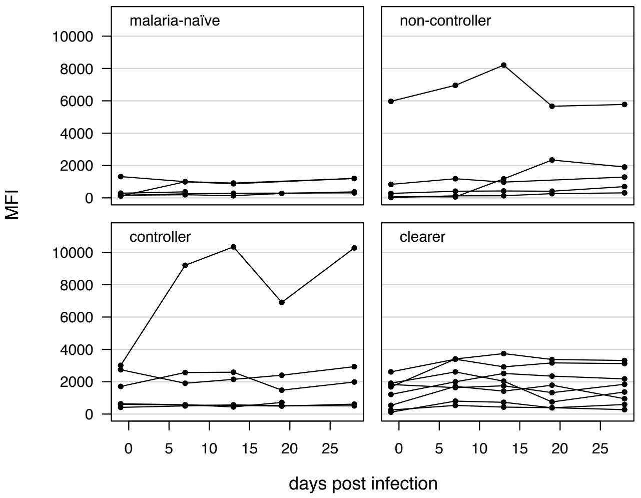

CIDRα1.8β_1

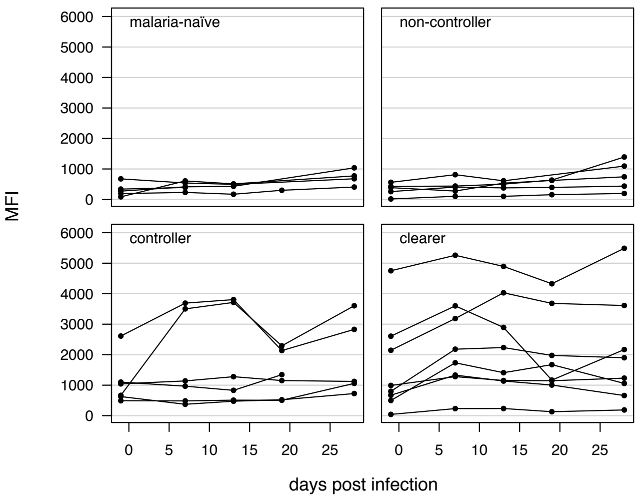

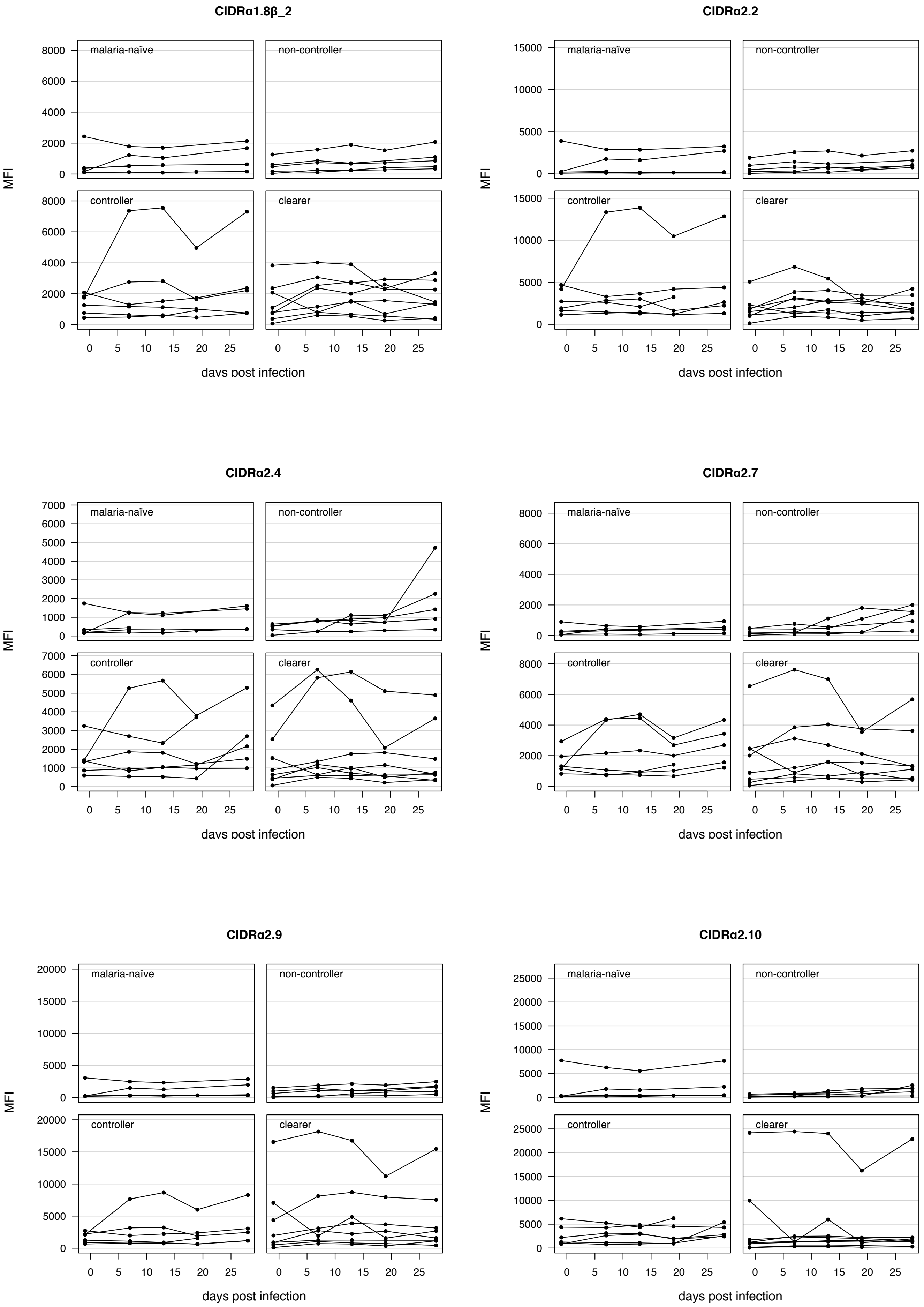
CIDRα1.8β_2
CIDRα2.2
CIDRα2.4
CIDRα2.7
CIDRα2.9
CIDRα2.10
malaria-naïve
non-controller
controller
clearer
MFI
days post infection

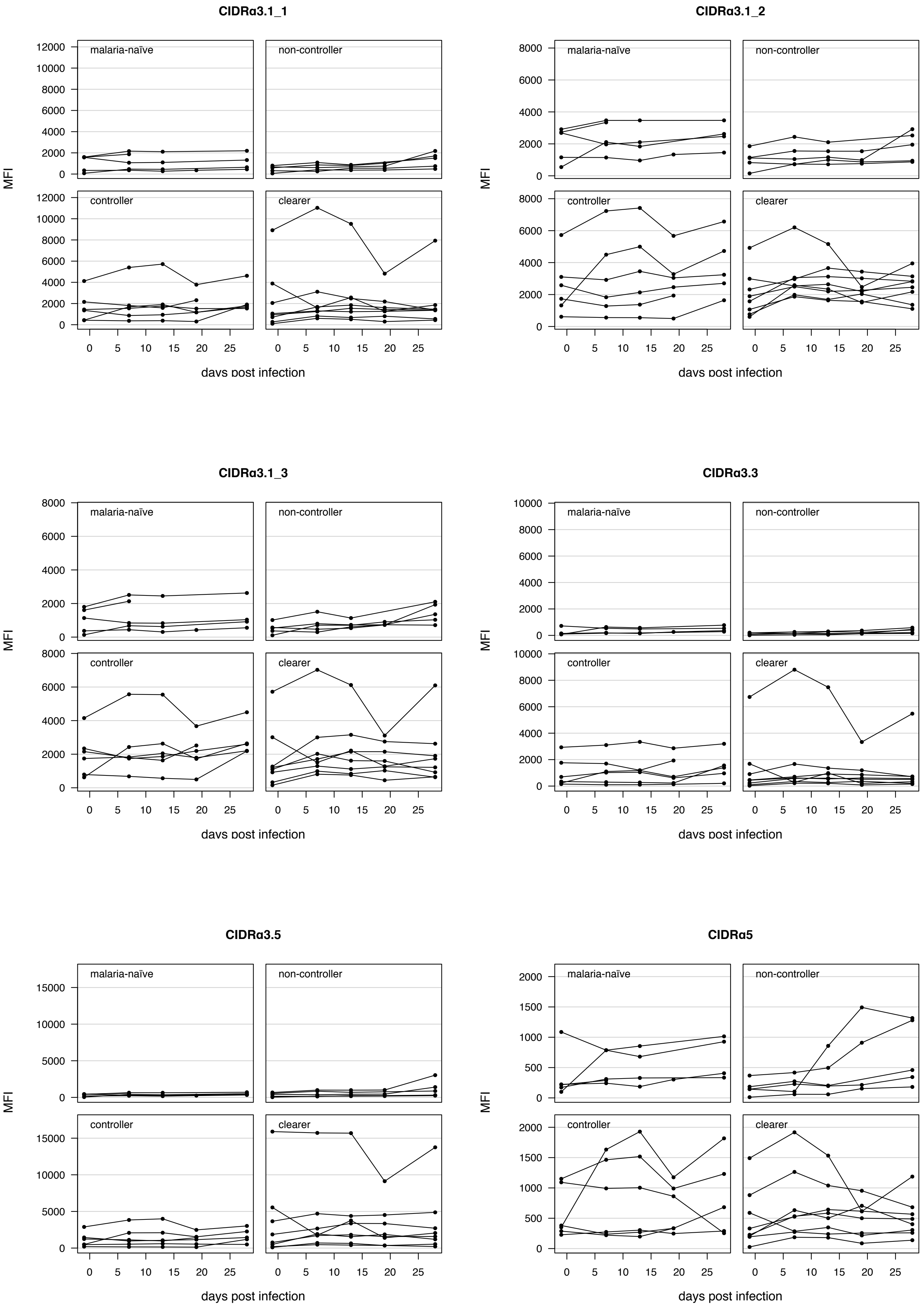
CIDRα3.1_1
CIDRα3.1_2
CIDRα3.1_3
CIDRα3.3
CIDRα3.5
CIDRα5
malaria-naïve
non-controller
controller
clearer
MFI
days post infection

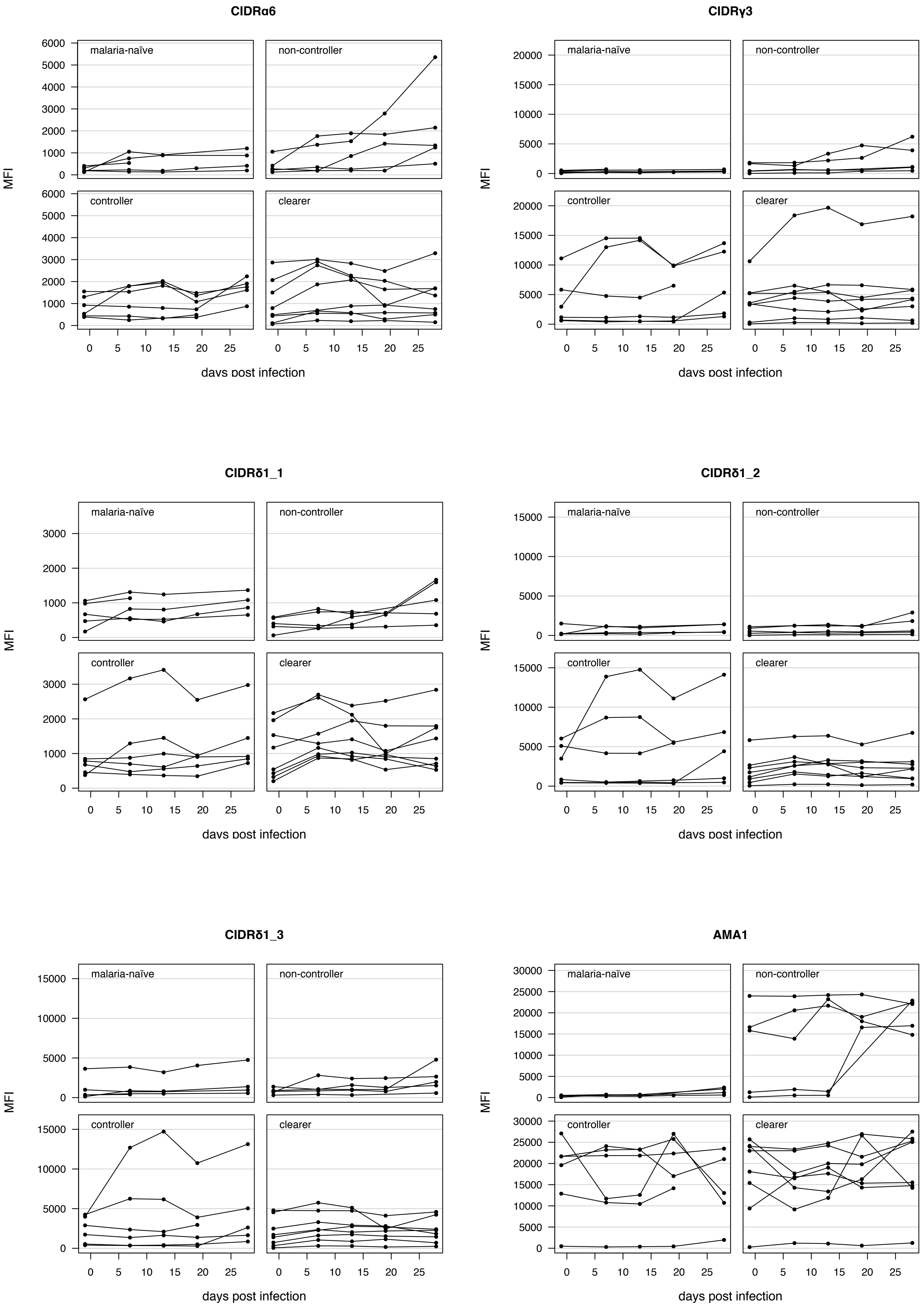
CIDRα6
CIDRγ3
CIDRδ1_1
CIDRδ1_2
CIDRδ1_3
AMA1
malaria-naïve
non-controller
controller
clearer
MFI
days post infection

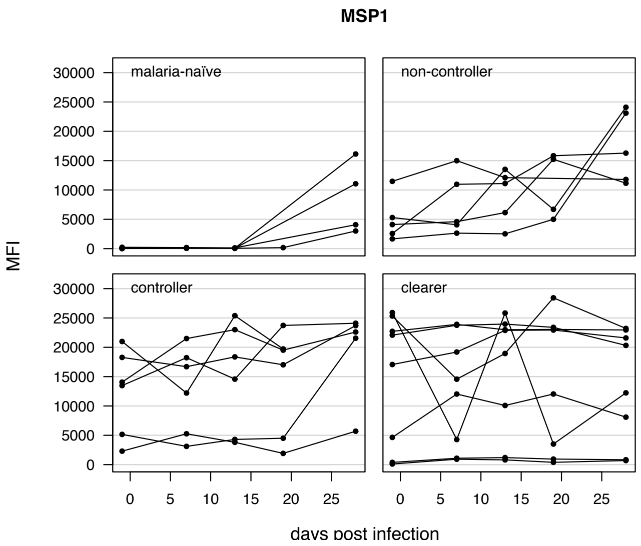
MSP1
malaria-naïve
non-controller
controller
clearer
MFI
0
5000
10000
15000
20000
25000
30000
0
5
10
15
20
25
days post infection

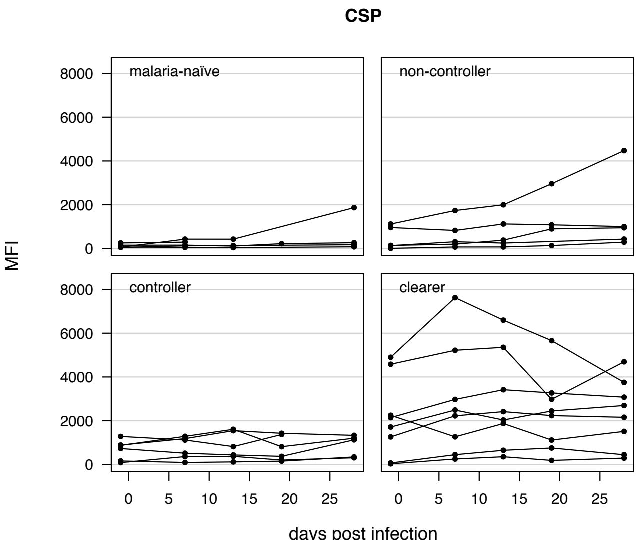
CSP
malaria-naïve
non-controller
controller
clearer
MFI
0
2000
4000
6000
8000
0
5
10
15
20
25
days post infection

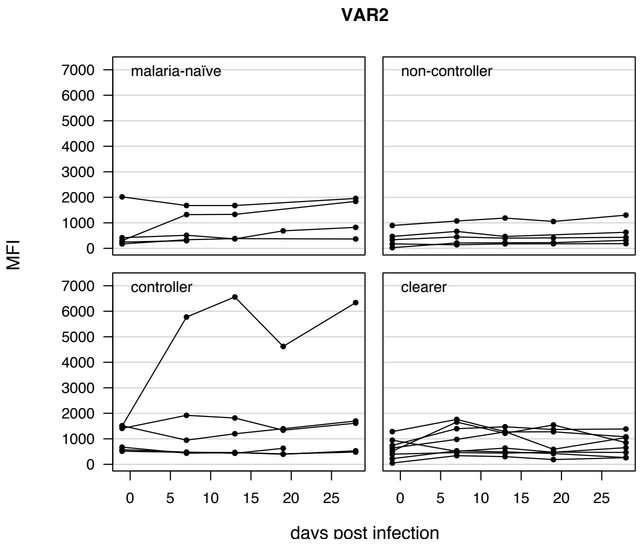
VAR2
malaria-naïve
non-controller
controller
clearer
MFI
0
1000
2000
3000
4000
5000
6000
7000
0
5
10
15
20
25
days post infection

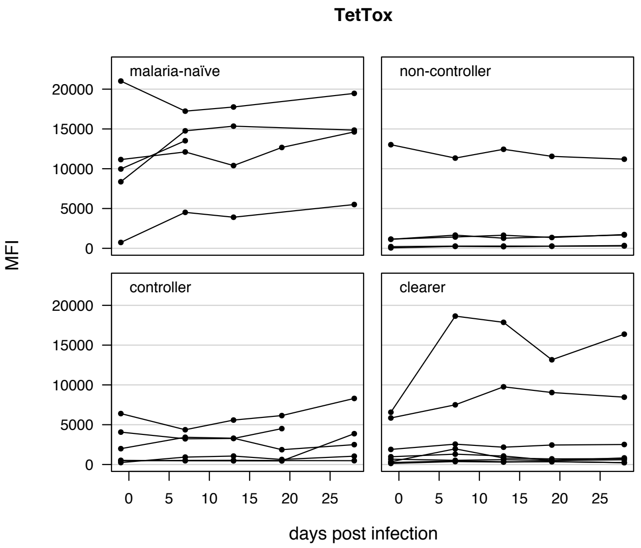
TetTox
malaria-naïve
non-controller
controller
clearer
MFI
0
5000
10000
15000
20000
0
5
10
15
20
25
days post infection

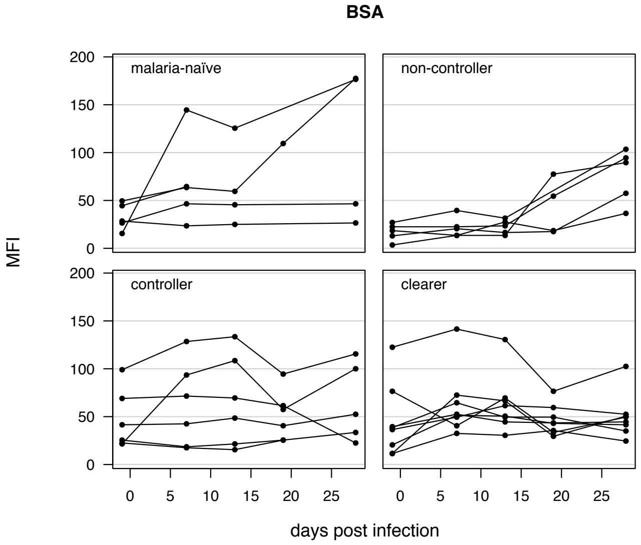
BSA
malaria-naïve
non-controller
controller
clearer
MFI
0
50
100
150
200
0
5
10
15
20
25
days post infection
